# The Influence of Different Classes of Amino Acids on Calcium Phosphates Seeded Growth

**DOI:** 10.3390/ma13214798

**Published:** 2020-10-27

**Authors:** Tea Mihelj Josipović, Monika Kovačević, Sarah Mateša, Marina Kostešić, Nives Matijaković, Borna Radatović, Daniel M. Lyons, Damir Kralj, Maja Dutour Sikirić

**Affiliations:** 1 Laboratory for Biocolloids and Surface Chemistry, Division of Physical Chemistry, Ruđer Bošković Institute, Bijenička Cesta 54, 10000 Zagreb, Croatia; tmihelj@irb.hr; 2 Faculty of Chemical Engineering and Technology, University of Zagreb, Marulićev Trg 19, 10000 Zagreb, Croatia; kovacevic.monika13@gmail.com (M.K.); marina.kostesic@gmail.com (M.K.); 3 Laboratory for Physical Chemistry of Aquatic Systems, Division for Marine and Environmental Research, Ruđer Bošković Institute, Bijenička Cesta 54, 10000 Zagreb, Croatia; Sarah.Matesa@irb.hr; 4 Laboratory for Precipitation Processes, Division of Materials Chemistry, Ruđer Bošković Institute, Bijenička Cesta 54, 10000 Zagreb, Croatia; Nives.Matijakovic@irb.hr (N.M.); kralj@irb.hr (D.K.); 5 Institute of Physics, Bijenička Cesta 46, 10000 Zagreb, Croatia; bradatovic@ifs.hr; 6 Center for Marine Research, Ruđer Bošković Institute, Giordano Paliaga 5, 52210 Rovinj, Croatia; lyons@cim.irb.hr

**Keywords:** octacalcium phosphate, dicalcium hydrogenphosphate dihydrate, amino acids, biomineralization, seeded growth

## Abstract

Amino acids (AAs) attract attention for elucidating the role of proteins in biomineralization and the preparation of functionalized biomaterials. The influence that AAs exert on calcium phosphate (CaP) mineralization is still not completely understood, as contradictory results have been reported. In this paper, the influence of the addition of different classes of AAs, charged (L-aspartic acid, Asp; L-lysine, Lys), polar (L-asparagine, Asn; L-serine, Ser; L-tyrosine, Tyr), and non-polar (L-phenylalanine, Phe), on CaP growth in the presence of octacalcium phosphate (OCP) and calcium hydrogenphosphate dihydrate (DCPD) seeds was investigated. In control systems (without AAs), a calcium-deficient apatite (CaDHA) layer was formed on the surface of OCP, while a mixture of CaDHA and OCP in the form of spherical aggregates was formed on the surface of DCPD crystals. Charged and non-polar promoted, while polar AAs inhibited CaDHA formation on the OCP seeds. In the case of DCPD, Lys, Asp, and Phe promoted CaP formation, while the influence of other AAs was negligible. The most efficient promotor of precipitation in both cases was non-polar Phe. No significant influence of AAs on the composition and morphology of precipitates was observed. The obtained results are of interest for understanding biomineralization processes and additive controlled material synthesis.

## 1. Introduction

Biomineralization, the formation of hard tissues in different organisms, is strictly regulated by an organic matrix and/or soluble biomolecules which facilitate the crystallization of inorganic phases and control their morphologies and nucleation sites [1,2]. Such tissues, due to their intricate architectures and hierarchical structures [3,4], possess specific mechanical properties often not achieved by manmade materials [5,6,7]. In addition, hard tissues are formed in an economical way, under mild chemical conditions in an aqueous environment at ambient or physiological temperature and pressure [1,2]. Therefore, it is not surprising that biomineralization continues to be an inspiration in materials science where emulation of such natural processes as biomimetic synthetic routes are now giving rise to what is considered green synthesis routes [8].

Among different biominerals, calcium phosphates (CaPs) attract special attention due to their role in both biological and pathological mineralization [1,9], as well as in a number of industrial processes [10,11]. CaPs appear in the form of different compounds among which in biomineralization, the most frequently occurring are amorphous calcium phosphate (ACP), octacalcium phosphate, (Ca_8_(HPO_4_)_2_(PO_4_)_4_·5H_2_O, OCP), calcium hydrogenphosphate dihydrate (CaHPO_4_·2H_2_O, DCPD), calcium-deficient hydroxyapatite (Ca_10−x_(HPO_4_)*_x_*(PO_4_)_6−x_(OH)_2−x_, 0 < x < 1, CaDHA) and hydroxyapatite (Ca_10_(PO_4_)_6_(OH)_2_, HA) [9,10]. While HA is the most frequently studied and used as a biomaterial due to its similarity to bone mineral, OCP, and DCPD are receiving increased attention both due to their proposed precursor role in HA formation, in vitro and in vivo [12,13,14,15,16,17,18,19], and as biomaterials for bone tissue regeneration [9,12,20].

Mineralization of carbonate apatite in bone is controlled by a framework of collagen fibers (extracellular matrix) and non-collagenous proteins [3,5]. Investigating the influence of amino acids (AAs) and protein building-blocks, on CaP formation and transformation was postulated both as a way to deepen the understanding of biomineralization [21,22,23], as well as a biomimetic route to improve CaP bioactivity [24,25].

Despite the existence of a significant number of studies on AAs’ influence on CaP formation and transformation, which are mostly focused on HA, their influence has still not been completely understood. Contradictory results have been reported in the literature, both regarding their inhibitory/induction effects on HA nucleation and growth [26,27,28] and their affinity towards HAP surfaces [27,29]. In addition, some studies were performed at higher pH to ensure that the AAs were completely dissociated and their net charge was negative. Considerably less work has been done with OCP [30] and DCPD [19,23,31].

Motivated by this lack of data, the influence of different AAs (L-aspartic acid, Asp; L-tyrosine, Tyr; L-asparagine, Asn; L-lysine, Lys; L-serine, Ser; and L-phenylalanine, Phe) on CaP formation initiated by OCP and DCPD seed crystals in metastable solution (MS) at pH 7.4 was investigated in this work. Seeded growth is considered to be of importance for biomineralization since it frequently involves heterogeneous nucleation [32], as for example, in bone formation during which calcium phosphates are formed on specific sites within the collagen matrix from metastable blood serum [33,34]. OCP and DCPD present good model systems for studying the interactions of organic molecules and inorganic crystals since they form well-developed crystals, with crystal faces of different ionic structure. Both of these CaP forms have a layered structure. The unit cell of OCP consists of alternating hydrated layers and layers closely resembling HA parallel to the (100) crystal plane [35,36,37]. In the case of DCPD, bi-layers of calcium and hydrogenphosphate ions, parallel to the (010) plane, alternate with bi-layers containing water molecules [38,39]. The growth morphology of both OCP and DCPD reflects their crystal structure, as plates with prominent (100) and (010) faces are formed, and at their surface hydrated layers are exposed in water [37,38]. The presence of a surface hydrate layer is important for interactions with different mineral ions and biomolecules as it can more easily adapt to the additive than a solid surface with a rigid position of ions, in which case successful interaction will occur if strict stereo compatibility exists [37,40,41]. In addition, hydrated surfaces have lower surface energy than non-hydrated surfaces [42,43]. At the applied experimental conditions, Asp is negatively and Lys positively charged, Asn, Ser, and Tyr are polar, while Phe is non-polar (Table S1). In addition, Ser and Asn are AAs that can form H-bonds.

## 2. Materials and Methods

### 2.1. Materials

Analytical grade chemicals and ultrapure water (18.2 MΩ∙cm; Merck Millipore “Milli-Q” system, Merck KGaA, Darmstadt, Germany) were used in all experiments. Calcium chloride dihydrate (CaCl_2_·2H_2_O) was purchased from Merck KGaA, while calcium hydroxide (Ca(OH)_2_), calcium acetate (Ca(CH_3_COO)_2_·H_2_O), sodium chloride (NaCl), hydrochloric acid (HCl), phosphoric acid (H_3_PO_4_), sodium hydrogenphosphate (Na_2_HPO_4_), sodium dihydrogen phosphate (NaH_2_PO_4_·H_2_O), L-lysine, and L-asparagine were obtained from Sigma−Aldrich, St. Louis, MO, USA. L-serine, L-phenylalanine, L-tyrosine, and L-aspartic acid were purchased from Alfa Aesar (Tewksbury, MA, USA).

OCP crystal seed was prepared by a slightly modified method proposed by LeGeros et al. [44,45]. In brief, 250 mL of 0.04 M Ca(CH_3_COO)_2_·H_2_O was added dropwise into 750 mL of a phosphate solution containing 5 mmol Na_2_HPO_4_·12H_2_O and 5 mmol NaH_2_PO_4_·H_2_O, for which the pH has been previously adjusted to 5 using concentrated HCl. The synthesis was carried out at 70 °C with slow mechanical stirring throughout 2 h (ca. 40 drops per min). The precipitate formation started after 30 min in the form of white flakes. After the reaction was completed, the precipitate was aged in mother liquor and stored for 24 h. Formed OCP crystals were separated by filtering through black ribbon filter paper (pore size 12–15 μm), repeatedly washed with Milli-Q water and ethanol, dried at 37 °C and kept in a desiccator.

DCPD seed crystals were synthesized by the reaction between Ca(OH)_2_ and H_3_PO_4_ to avoid incorporation of cationic or anionic impurities in the crystal structure by the previously described procedure [46]. In brief, calcium hydroxide stock solution was prepared by the addition of an excess of calcium hydroxide to Milli-Q water bubbled with N_2_ and subsequent filtering of the suspension through a 0.22 μm membrane filter. The saturated solution was kept under an N_2_ atmosphere to prevent contamination with CO_2_. Reactant solutions were prepared by diluting concentrated stock solutions to *c*(Ca(OH)_2_) = *c*(H_3_PO_4_) = 0.02 mol dm^−3^. The DCPD precipitation was initiated by pouring the phosphate solution into an equal volume of calcium solution. The synthesis was performed in a thermostated double-walled glass vessel with a 400 cm^3^ capacity at 25 °C with constant magnetic stirring. To minimize the exchange of carbon dioxide between the ambient air and the reaction system the vessel was tightly closed. After a 1 h reaction time, the obtained precipitate was filtered through a 0.22 μm membrane filter, thoroughly washed with small portions of Milli-Q water and ethanol, dried in a stream of nitrogen, and kept in a desiccator.

### 2.2. Precipitation Experiments

#### 2.2.1. Preparation of Stock Solutions

CaCl_2_, Na_2_HPO_4_, and AA stock solutions were prepared by dissolving the required amount of chemicals in 0.15 mol dm^−3^ NaCl solution. Chemicals were dried overnight in a desiccator over silica gel. HCl was used for adjusting the pH of the sodium hydrogenphosphate stock solution. All stock solutions contained 0.15 mol dm^−3^ NaCl.

#### 2.2.2. Preparation of Metastable Solutions

In order to follow interactions of AAs with crystals seeds, a metastable solution (MS), that is, a solution in which no observable precipitation occurred during a 4 h reaction time, was prepared by mixing equal volumes (*V* = 20 mL) of equimolar calcium chloride and sodium hydrogenphosphate solutions and 0.5 mL Milli-Q water (to account for the addition of crystal seed suspension). Initial concentrations of reactants in the metastable solutions were *c*(CaCl_2_) = *c*(Na_2_HPO_4_) = 0.004198 mol dm^−3^ and *c*(NaCl) = 0.148 mol dm^−3^ to maintain the ionic strength. The metastable solutions containing 5 or 10 ppm AAs were prepared in the same manner using anionic reactant solutions containing the required amount of AAs.

#### 2.2.3. Precipitation Systems

All experiments were performed in a thermostated double-walled glass vessel with a 45 cm^3^ capacity at 25 °C with constant magnetic stirring. Suspensions of 1 mg of crystal seed in 0.5 mL of Milli-Q water were freshly prepared before each experiment using an ultrasonic bath for 10 min. The crystal seed suspension was quickly added to 40 mL of metastable solutions. The system without added AAs is denoted as the control system (CS). The advancement of the precipitation process was monitored by continuously monitoring pH changes (SI Analytics Lab 845 pH/ion meter, Xylem Analytics, Mainz, Germany). For each precipitation system, the measurements were repeated five times. Based on pH measurements, samples for further analysis were taken after an abrupt pH drop and after a 4 h reaction time to enable precipitate characterization at different stages of the process.

### 2.3. Precipitate Characterization

Precipitates were filtered through a 0.22 μm Millipore filter at predetermined time intervals, washed three times with Milli-Q water and once with ethanol, dried in a stream of nitrogen. Samples were kept in a desiccator until further analysis.

PXRD patterns were collected by Rigaku Ultima IV diffractometer (Rigaku, Tokyo, Japan), operating at 40 kV and 40 mA, in Bragg-Brentano parafocusing geometry using CuKα radiation and 5° Soller slits. Samples were scanned in the range from 3.25 to 60° 2*θ* in 0.02° steps with a scan speed of 1° min^−1^.

FTIR spectra were obtained with an FTIR spectrometer equipped with an attenuated total reflection module (Tensor I, Bruker, Ettlingen, Germany). Scan range from 4000–400 cm^−1^, with a resolution of 2 cm^−1^ was used. The obtained spectra are the average of 32 scans.

The morphology of the obtained precipitates was observed by scanning electron microscopy (SEM) on a JEOL Field Emission JSM-7000F 7000F (JEOL, Tokyo, Japan)and TESCAN tungsten filament VEGA3 microscopes (Tescan, Brno—Kohoutovice, Czech Republic). Image acquisition was done at 10 mm working distance, 5 kV acceleration voltage, and 5000× and 10,000× magnifications.

## 3. Results and Discussion

### 3.1. Influence of Amino Acids on the Rate of Precipitation

The interactions of different AAs with OCP and DCPD crystals were investigated in a slightly supersaturated, metastable, solution, in which no observable precipitation occurs in the absence of crystal seed. Precipitation of calcium phosphates is followed by a decrease in the pH of the system, which enables at least semiquantitative monitoring of the progress of the reaction by following changes in pH [47,48,49,50]. Typically, sigmoidal shaped pH vs. time curves, reflecting the different stages of the precipitation process, were observed (Figures S1 and S2). The initial small or negligible change in pH (stage I) is considered to reflect, depending on experimental conditions, either induction time for the commencement of solid phase formation (direct crystallization, seeded growth) or formation of an amorphous phase (spontaneous precipitation in neutral or basic solutions). The subsequent abrupt decrease in pH (stage II) reflects the precipitation of a crystalline phase (upon crystal seeds or amorphous phase). The final slight change in pH (stage III) is associated with solution-mediated growth and phase transformation [47,48,49,50]. Therefore, to confirm that during the reaction time no spontaneous precipitation occurs in metastable solution, pH was continuously followed with or without added AAs. No pH changes were detected in any of the metastable solutions except those containing 5 ppm of Ser and 10 ppm of Phe (Figure S1). In the presence of Ser, precipitation started after 138 min, while in presence of Phe, after 71 min. In both systems poorly crystalline CaDHA was formed after a 4 h aging time (Figure S3). Similar results were found by Jahormi and Cerruti [51], who have shown that calcium and phosphate ions can interact with glutamic acid and arginine to form aggregates which, if undisturbed, grow and give rise to HA. Adding either OCP or DCPD seed crystals initiated the precipitation as evidenced by the decrease in pH. The time needed for the commencement of precipitation, induction time (t_ind_), was similar for both seed crystals (Table 1). However, AAs significantly influenced induction times depending both on their concentration, as well as on the type of seed crystal used. At higher concentrations, both charged AAs, Asp and Lys, reduced the induction time in the presence of OCP seed crystals by almost the same value. Conversely, at lower concentration, Lys was found to inhibit precipitation. Polar amino acids acted as inhibitors in the presence of OCP seed crystals, with Ser being the most efficient inhibitor. Surprisingly, the most efficient promotor was the non-polar aromatic Phe. It was previously observed that during spontaneous precipitation of HA, addition of Asp, Gly, and Lys induced ACP transformation to HA [28], while Glu and Arg inhibited HA formation [21].

In the presence of DCPD seed crystals, no significant inhibition of seeded growth was observed. The influence of Asp was negligible, as previously shown for spontaneous precipitation [31], while Lys reduced the induction time only at higher concentrations, almost to the same extent as in the presence of OCP seeds. On the contrary, Chu et al. [19] have shown that in phosphate solution, acidic AAs, Asp, and Glu promote HA crystallization from DCPD seeds by reducing the interfacial energy barrier between these two phases. The observed difference in the behavior of Asp observed in this study, could be a consequence of the formation of the Asp/Ca^2+^ complexes [21] which significantly reduced the concentration of free Ca^2+^ and consequently the supersaturation. Among polar AAs, Asn was the most effective in promoting seeded growth on DCPD crystals, in contrast to Ser, which had the largest influence on OCP seeded growth. Yet again, nonpolar Phe was the most efficient in promoting precipitation. Considering that at the higher Phe concentration, precipitation of CaDHA was initiated in metastable solution, it seems plausible that Phe aggregates indeed have been formed in MS, similar to the behavior of polar Glu and Arg [51]. It should be stressed that under our experimental conditions both α-COOH and α-NH_2_ groups of Phe are charged (Table S1). The difference in the observed AAs’ effect on the kinetics of crystal growth of OCP and DCPD crystals could point to the difference in the mechanism of crystal growth for different seed crystals. Indeed, it is known that OCP transformation to HAP can proceed via the dissolution-reprecipitation mechanism or by topotaxial conversion by ion diffusion within the crystal lattice [52], while for DCPD, a transformation dissolution—reprecipitation mechanism is proposed [19].

Based on pH vs. time curves, precipitates for further structural and morphological analysis were taken at the beginning of stage III, as well as after 240 min. The beginning of stage III corresponded to different aging times in the presence of OCP seed crystals (60 min for Asp, Lys and Phe; 90 min for Asn and Tyr; and 120 min for Ser) and 60 min aging time in the presence of DCPD seed crystals.

### 3.2. Influence on Composition and Morphology

**Figure 1:**
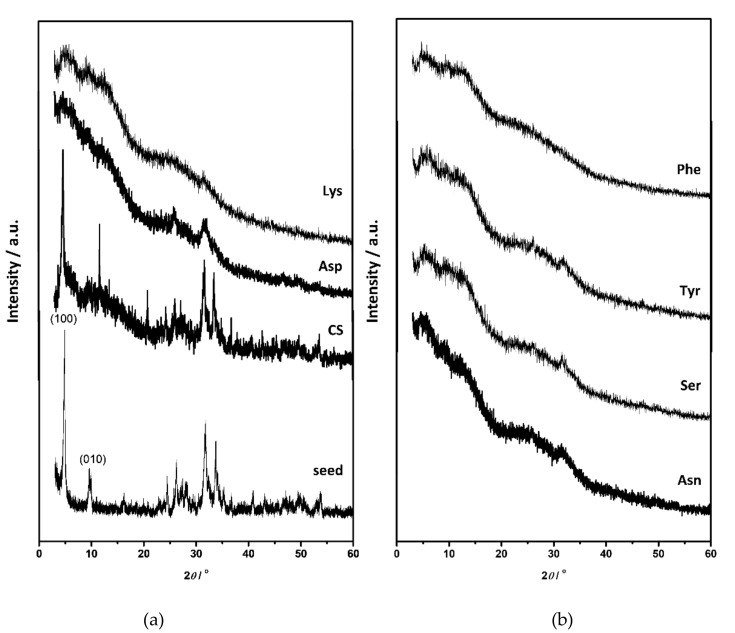
PXRD diffractograms of (**a**) octacalcium phosphate (OCP) seed crystals and precipitates formed in the control system (CS) and in the presence of aspartic acid (Asp) and lysine (Lys), (**b**) precipitates obtained in the presence of asparagine (Asn), serine (Ser), tyrosine (Tyr) and phenylalanine (Phe) after aging time corresponding to commencement of stage III in pH vs. time curves in the system *c(*CaCl_2_) = *c*(Na_2_HPO_4_) = 4.198 mmol dm^−3^, *c*(NaCl) = 0.148 mol dm^−3^, *m*(seed OCP) = 1 mg, *γ*(AA) = 10 ppm, except *γ*(Phe) = 5 ppm. *t* = 25 °C, pH_initial_ = 7.4, magnetic stirring.

**Figure 2:**
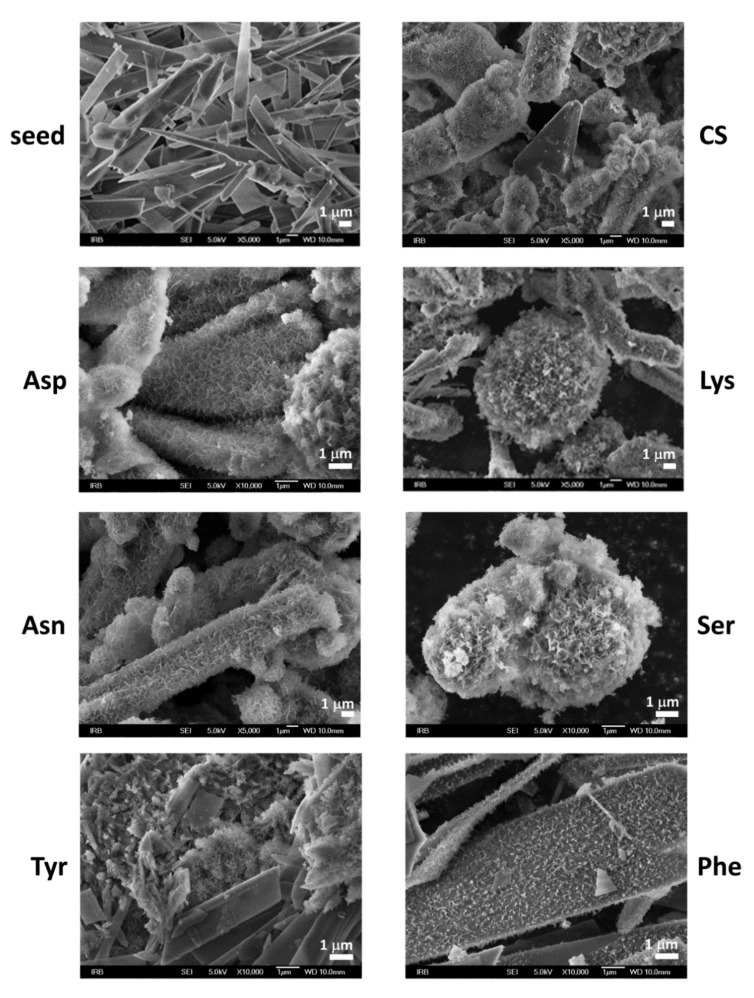
SEM micrographs of octacalcium phosphate (OCP) seed crystals as well as precipitates formed in the control system (CS) and in the presence of different amino acids after aging time corresponding to commencement of stage III in pH vs. time curves in the system *c*(CaCl_2_) = *c*(Na_2_HPO_4_) = 4.198 mmol dm^−3^, *c*(NaCl) = 0.148 mol dm^−3^, *m*(seed OCP) = 1 mg, *γ*(AA) = 10 ppm, except *γ*(Phe) = 5 ppm. *t* = 25 °C, pH_initial_ = 7.4, magnetic stirring. Asp—L-aspartic acid, Tyr—L-tyrosine, Asn—L-asparagine, Lys—L-lysine, Ser—L-serine, Phe—L-phenylalanine.

The composition and structure of OCP seed crystals were confirmed by PXRD and FTIR analysis. PXRD diffractograms of OCP seed crystals (Figure 1a, Table S2) comprised a number of sharp peaks, with the most intense noted at 4.86°, 9.42°, 9.89°, 26.2°, and 31.68° 2*θ* corresponding to (100), ($\bar{1}$10), (010), ($\bar{1}$02), and ($\bar{4}$02) reflections, respectively. The FTIR spectrum encompassed phosphate and water bands characteristic for OCP (Figure S4 and Table S3). The bands characteristic for water, that is, stretching and bending of OH groups, were detected in the 3619–2579 cm^−1^ region as well as at 1636 cm^−1^, respectively. In the region 1293–957 cm^−1^ the vibrational bands of phosphate groups with hyperfine structure were observed. The -P-(OH)- stretching vibrations were detected at 915 cm^−1^ and 856 cm^−1^. In addition, ν_4_ and ν_2_PO_4_ bands were detected at 602 cm^−1^ and 453 cm^−1^ respectively, while ν_4_HPO_4_ bands were observed at 560 cm^−1^ and 525 cm^−1^ [53,54]. The OCP seed crystals appeared in a form of well-separated, thin, blade-like crystals (Figure 2) [44].

When suspended in water or phosphate solution, OCP crystals readily transform to HA [15,55,56,57]. Two mechanisms were proposed for this process, in-situ hydrolysis or OCP dissolution followed by HA precipitation [52,57]. However, if OCP seed crystals are suspended in CaP saturated solution, the formation of different CaP phases and the simultaneous dissolution of OCP occurs [52]. The PXRD pattern of the precipitates formed in the control system at the beginning of stage III still shows the most prominent OCP reflections (Figure 1). However, in the FTIR spectrum the hyperfine structure of phosphate bands in the region 1293–957 cm^−1^ is no longer observed, pointing to the ongoing formation of a new phase on the OCP seed crystals (Figure S4). Changes in the PXRD patterns of precipitates formed in the presence of AAs are more pronounced. Low crystalline precipitates were obtained in all cases. The PXRD diffractograms of precipitates formed in the presence of Asp, Asn, Tyr, and Ser showed peaks at around 26.00° 2*θ* characteristic of CaDHA (Table S2) [58]. The PXRD diffractogram of precipitates formed in the presence of Phe, in addition to the low intensity ($\bar{1}$10) OCP reflection, as well as that at 22.61° 2*θ*, showed only an amorphous maximum in the region 20–32° 2*θ* characteristic of ACP [58]. It is interesting to note that in the presence of the amino acids the ratio of the intensities of the phosphate bands at around 1285 cm^−1^ and at around 557 cm^−1^, as well as the ratio of intensities of the bands at around 1022 cm^−1^ and at around 557 cm^−1^, are lower than observed for the OCP seed and in the control system (Table S4). SEM micrographs of the precipitates formed at the beginning of stage III (Figure 2), revealed the formation of layers of leaf-like crystals typical of CaDHA on the surface of the OCP crystals [46]. Only small amounts of spherical aggregates of leaf-like CaDHA crystals, not grown on the surface of OCP crystals, were detected. No significant differences in morphology of CaDHA crystals were observed in the presence of different AAs. However, the smallest coverage of OCP crystals was observed in the presence of Tyr and Phe.

**Figure 3:**
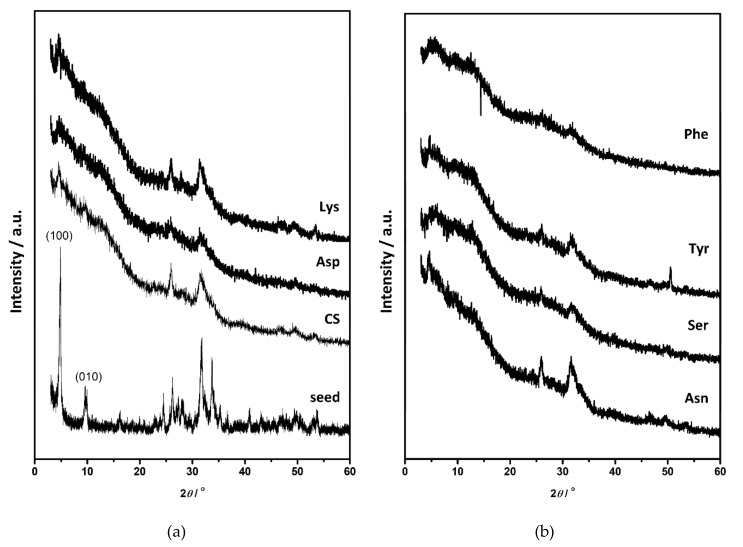
PXRD diffractograms of (**a**) octacalcium phosphate (OCP) seed crystals and precipitates formed in the control system (CS) and in the presence of aspartic acid (Asp) and lysine (Lys), (**b**) precipitates obtained in the presence of asparagine (Asn), serine (Ser), tyrosine (Tyr) and phenylalanine (Phe) after 240 min aging time in the system in *c(*CaCl_2_) = *c*(Na_2_HPO_4_) = 4.198 mmol dm^−3^, *c*(NaCl) = 0.148 mol dm^−3^, *m*(seed OCP) = 1 mg, *γ*(AA) = 10 ppm, except *γ*(Phe) = 5 ppm. *t* = 25 °C, pH_initial_ = 7.4, magnetic stirring.

**Figure 4:**
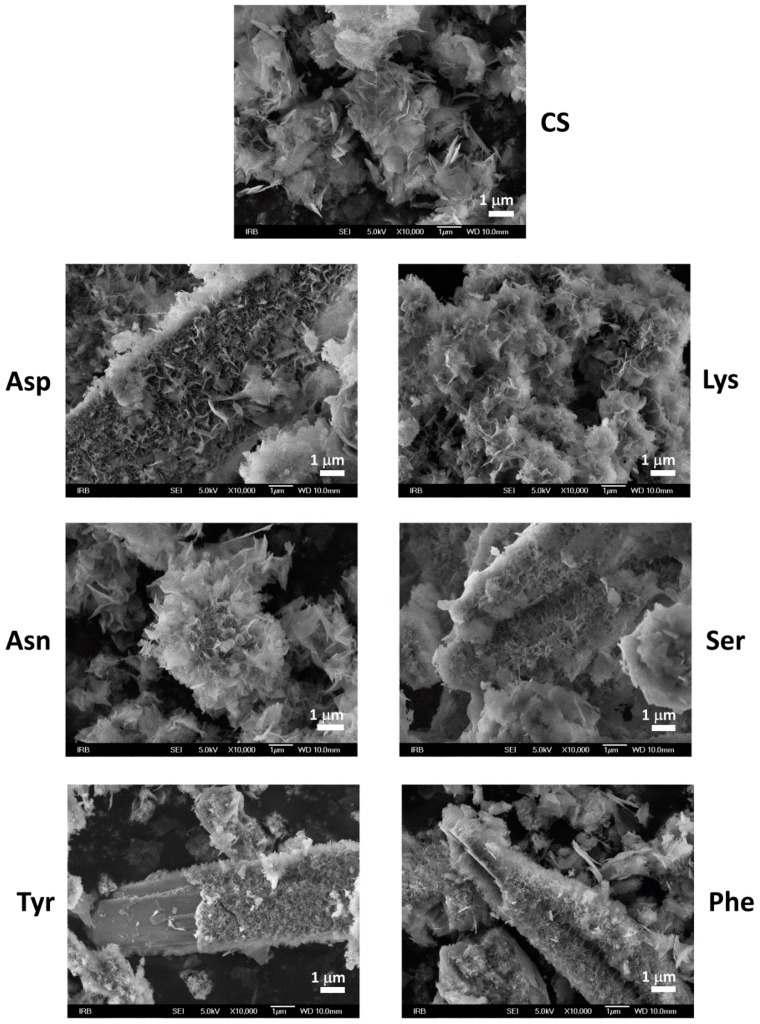
SEM micrographs of precipitates formed in the control system (CS) and in the presence of different amino acids after 240 min aging time in the system *c(*CaCl_2_) = *c*(Na_2_HPO_4_) = 4.198 mmol dm^−3^, *c*(NaCl) = 0.148 mol dm^−3^, *m*(seed OCP) = 1 mg, *γ*(AA) = 10 ppm, except *γ*(Phe) = 5 ppm. *t* = 25 °C, pH_initial_ = 7.4, magnetic stirring. Asp—L-aspartic acid, Tyr—L-tyrosine, Asn—L-asparagine, Lys—L-lysine, Ser—L-serine, Phe—L-phenylalanine.

After 240 min the changes were more pronounced. In addition to characteristic CaDHA reflections, the (100) reflection of OCP was visible in the PXRD patterns of precipitates formed in the control system and in the presence of charged Asp and Lys, as well as polar Asn, but not in the presence of other investigated AAs (Figure 3, Table S5). The least crystalline precipitate was formed in the presence of Phe. The OCP crystals covered with CaDHA crystals could still be seen after 240 min (Figure 4) and the thickness of the surface layer, surface coverage as well as the amount of aggregates of leaf-like CaDHA crystals not grown on the surface of OCP crystals increased in comparison to the beginning of stage III. In addition, the difference in the ratio of the intensities of phosphate bands of the precipitates formed in the presence of amino acids and in the control system is not so pronounced after 1 h (Figure S5, Table S4 and S6).

The observed formation of CaDHA on the surface of OCP seed crystals is consistent with a previous study published by Nancollas et al. [55]. They have shown that at pH 7.4, ACP is the first solid phase to form on the surface of OCP crystals, which subsequently transforms into a poorly crystalline phase, with HA forming after several weeks of aging.

**Figure 5:**
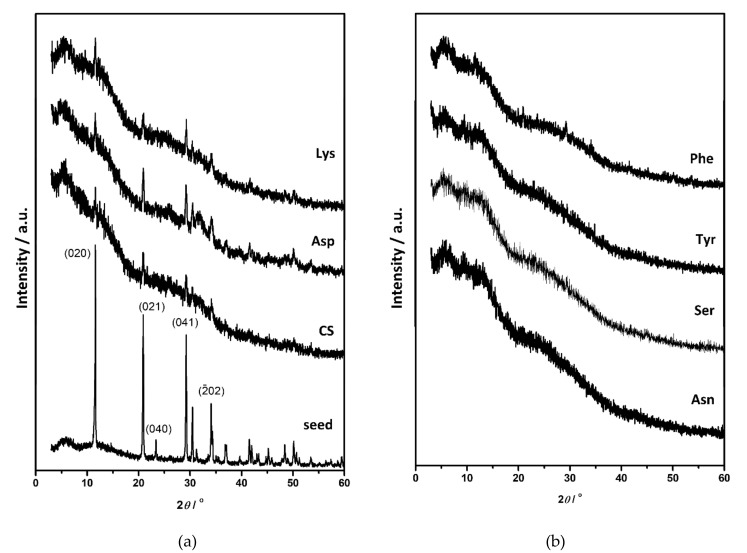
PXRD diffractograms of (**a**) calcium hydrogenphosphate dihydrate (DCPD) seed crystals and precipitates formed in the control system (CS) and in the presence of aspartic acid (Asp) and lysine (Lys), (**b**) precipitates obtained in the presence of asparagine (Asn), serine (Ser), tyrosine (Tyr) and phenylalanine (Phe) after 60 min aging time in the system *c*(CaCl_2_) = *c*(Na_2_HPO_4_) = 4.198 mmol dm^−3^, *c*(NaCl) = 0.148 mol·dm^−3^, *m*(seed DCPD) = 1 mg, *γ*(AA) = 10 ppm, except *γ*(Phe) = 5 ppm. *t* = 25 °C, pH_initial_ = 7.4, magnetic stirring.

**Figure 6:**
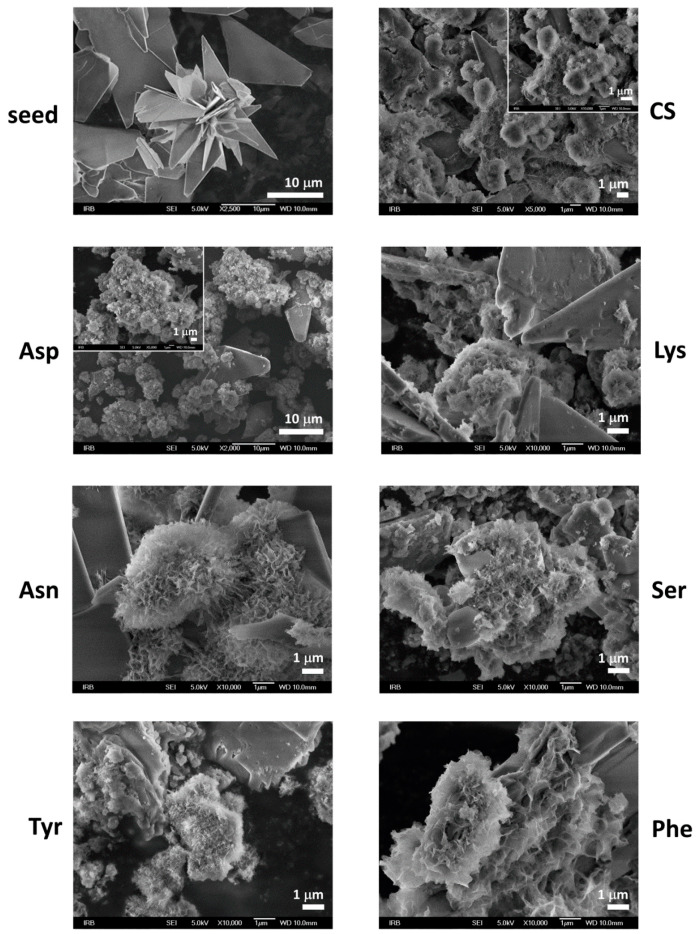
SEM micrographs of calcium hydrogenphosphate dihydrate (DCPD) seed crystals as well as precipitates formed in the control system (CS) and in the presence of different amino acids after 60 min aging time in the system *c*(CaCl_2_) = *c*(Na_2_HPO_4_) = 4.198 mmol dm^−3^, *c*(NaCl) = 0.148 mol dm^−3^, *m*(seed DCPD) = 1 mg, *γ*(AA) = 10 ppm, except *γ*(Phe) = 5 ppm. *t* = 25 °C, pH_initial_ = 7.4, magnetic stirring. Asp—L-aspartic acid, Tyr—L-tyrosine, Asn—L-asparagine, Lys—L-lysine, Ser—L-serine, Phe—phenylalanine.

The PXRD pattern of DCPD seed crystals showed characteristic diffraction peaks at 11.58°, 20.91°, 29.22°, and 34.08° 2*θ* corresponding to the (020), (021), (041), and ($\bar{2}$20) reflections (Figure 5a, Table S7). In the FTIR spectrum water vibrations in the 3600–3000 cm^−1^ region, with superimposed bands at 3548 cm^−1^, 3493 cm^−1^, 3291 cm^−1^, and 3149 cm^−1^, as well as a band at 1636 cm^−1^ were observed, with hydroxyl and phosphate bands in the region 1230–1000 cm^−1^ and at 987 cm^−1^, characteristic for DCPD (Figure S6, Table S8) [59,60]. SEM micrographs showed that typical, large thin, plate-like DCPD crystals were obtained (Figure 6), which is in accordance with previous studies [23,31,39].

Similar to OCP, DCPD transforms to CaDHA and/or HA when suspended in water and electrolyte solutions [16,61,62]. PXRD diffractograms (Figure 5) and FTIR spectra (Figure S6) of the precipitates obtained in DCPD seeding experiments after 60 min of aging confirm that DCPD transformation had started. The main DCPD reflections, corresponding to the (020), (021), (041), and ($\bar{2}$20) planes, were of low intensity in PXRD patterns of the precipitates formed in the CS and in the presence of AA. In addition, the reflections at 25.87° and/or 31.62° 2*θ* characteristic for CaDHA [58] were observed in the diffractograms of precipitates formed in the presence of Asp and Lys. In the FTIR spectra of all samples (Figure S6) the hyper-fine structure of the bands in the region 1250–1000 cm^−1^ is no longer observed, confirming an ongoing transformation. In addition, the intensity ratios of the phosphate bands at around 872 cm^−1^ and at around 560 cm^−1^, are higher while ratios of the bands at around 560 cm^−1^ and 525 cm^−1^ are lower in the presence of amino acids as compared with the control system (Table S9). SEM micrographs (Figure 6) revealed that the new phase dominantly precipitated in the form of spherical aggregates of small leaf-like crystals that are typical for CaDHA [46]. This is in contrast with OCP seeding, in which CaDHA dominantly formed layers on the surface of OCP crystals. In addition, pits seen on the bare surfaces of DCPD crystals could point to DCPD dissolution.

**Figure 7:**
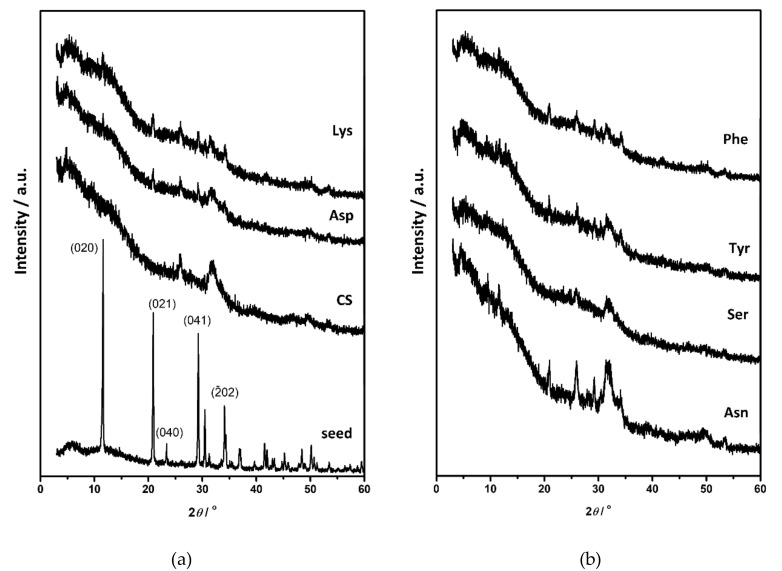
PXRD diffractograms of (**a**) calcium hydrogenphosphate dihydrate (DCPD) seed crystals and precipitates formed in the control system (CS) and in the presence of aspartic acid (Asp) and lysine (Lys), (**b**) precipitates obtained in the presence of asparagine (Asn), serine (Ser), tyrosine (Tyr) and phenylalanine (Phe) after 240 min aging time in the system *c*(CaCl_2_) = *c*(Na_2_HPO_4_) = 4.198 mmol dm^−3^, *c*(NaCl) = 0.148 mol dm^−3^, *m*(seed DCPD) = 1 mg, *γ*(AA) = 10 ppm, except *γ*(Phe) = 5 ppm. *t* = 25 °C, pH_initial_ = 7.4, magnetic stirring.

**Figure 8:**
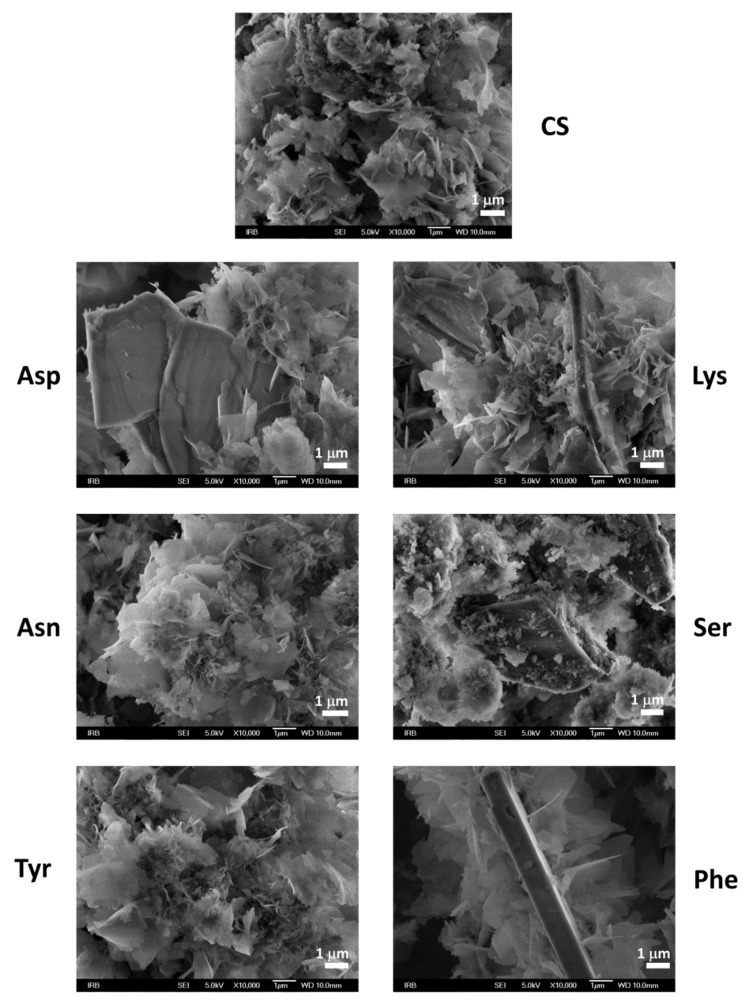
SEM micrographs of precipitates formed in the control system (CS) and in the presence of different amino acids after 240 min aging time in the system *c*(CaCl_2_) = *c*(Na_2_HPO_4_) = 4.198 mmol dm^−3^, *c*(NaCl) = 0.148 mol dm^−3^, *m*(seed DCPD) = 1 mg, *γ*(AA) = 10 ppm, except *γ*(Phe) = 5 ppm. *t* = 25 °C, pH_initial_ = 7.4, magnetic stirring. Asp—L-aspartic acid, Tyr—L-tyrosine, Asn—L-asparagine, Lys—L-lysine, Ser—L-serine, Phe—L-phenylalanine.

The changes became more pronounced after 240 min. In the PXRD patterns (Figure 7, Table S10) of all samples, except precipitates formed in the presence of Lys and Phe, low-intensity OCP (100) and/or ($\bar{1}$10) reflections appeared in addition to DCPD reflections. All PXRD patterns contained the most prominent CaDHA reflection at around 31.90° 2*θ*, as well as some CaDHA low angle reflections [58,63]. FTIR spectra (Figure S7, Table S11) of the obtained precipitates showed phosphate bands corresponding to the asymmetric stretching mode of PO_4_^3−^ at around 1113 cm^−1^ and 1020 cm^−1^, symmetric stretching at around 960 cm^−1^, and bending modes of PO_4_^3−^ at around 600 cm^−1^, 560 cm^−1^, and 526 cm^−1^. Bands corresponding to water vibrations were found at around 3700–2500 cm^−1^ and at around 1640 cm^−1^ [59,60]. Only in spectra of the precipitates obtained in the presence of Lys, low-intensity DCPD water bands at 3530 and 3471 cm^−1^ were observed. The difference in intensity ratios of bands at around 872 cm^−1^ and at around 560 cm^−1^ is lower than after 1 h aging time, and the difference between the precipitates formed in the presence of amino acids and in the control system is not so pronounced as after 1 h (Table S9). PXRD and FTIR data point to the formation of a mixture of OCP and CaDHA on the surface of DCPD crystals in the presence of all investigated AAs except Lys and Phe. This was confirmed by SEM micrographs (Figure 8) in which individual DCPD crystals are still seen with spherical aggregates of leaf-like crystals, and which are much bigger than those obtained after 60 min aging time. No significant differences in morphology of formed precipitates obtained in the presence of different AAs have been observed.

The literature data for DCPD seeded growth show, at first glance, contradictory results. Nancollas and Wefel [55] have shown that by introducing DCPD into solution supersaturated to DCPD, OCP, TCP, and HA (*c*(Ca^2+^) = 1.4 mmol dm^−3^, *c*(PO_4_^3−^) = 0.8 mmol dm^−3^) at pH 7.4 no significant CaP precipitation was achieved. However, when simulated body fluid (SBF) was inoculated with Na- and K-doped DCPD, it completely transformed into HA in less than one week [62]. These findings could indicate that a critical supersaturation point for CaP growth on DCPD seed crystals at physiological pH exists and that it is higher than that for OCP seed crystals. In addition to experimental conditions [16], this process could also be influenced by additives. Thus, calcium ions can accelerate DCPD transformation to HA [63]. Acidic AAs also accelerate HA crystallization from DCPD, unlike non-acidic AAs. The observed difference was attributed to the ability of acidic AAs to reduce the interfacial energy barrier between DCPD and HAP [19]. However, in their recent study, Rubini et al. [23] showed that Asp is not incorporated into DCPD crystals during synthesis, nor does it influence either its crystallization or hydrolysis, although when present in a solution, Asp delays DCPD transformation into more stable phases.

## 4. Conclusions

The influence of different AAs, encompassing charged Asp and Lys, polar Asn, Ser and Tyr, and non-polar Phe, on calcium phosphate growth, initiated with OCP and DCPD crystal seeds in metastable solution at physiological pH was investigated. It was shown that the influence of AAs on the rate of seeded growth depends on the type of seed applied. The observed effects ranged from inhibition to promotion in the case of OCP and only promotion in the case of DCPD seed. Surprisingly, in both cases, non-polar Phe showed the strongest promotion effect which was attributed to the formation of its aggregates in a solution. No effect of AAs on the composition and morphology of CaDHA formed on OCP crystals was observed. CaDHA precipitated in the form of leaf-like crystal layers on the OCP crystals. On the contrary, when using DCPD crystals, spherical aggregates of leaf-like crystals formed. PXRD and FTIR analysis confirmed that, except in the presence of Lys and Phe, a mixture of CaDHA and a small amount of OCP was formed. In addition to contributing to the understanding of biomineralization processes, the results obtained herein may be of interest for the preparation of functionalized CaP materials as well.

## Figures and Tables

**Table 1:** Average induction times (*t*_ind_) obtained from pH vs. t curves (Figure S1) from five measurements expressed with standard deviation. *c*(CaCl_2_) = *c*(Na_2_HPO_4_) = 4.198 mmol dm^−3^, *c*(NaCl) = 0.148 mol dm^−3^, *m*(seed) = 1 mg, pH_i_ = 7.4, *t* = 25 °C, magnetic stirring. Asp—L-aspartic acid, Tyr—L-tyrosine, Asn—L-asparagine, Lys—L-lysine, Ser—L-serine, Phe—L-phenylalanine.

Amino Acid	γ/ppm	*t*_ind_/min	
		OCP Seed	DCPD Seed
Asp	0	46.90 ± 3.34	44.68 ± 2.39
	5	31.47 ± 3.24	42.29 ± 2.90
	10	34.65 ± 2.62	47.38 ± 2.98
Lys	5	65.17 ± 4.51	47.12 ± 2.15
	10	34.36 ± 3.15	31.33 ± 2.95
Asn	5	56.31 ± 4.23	39.77 ± 0.96
	10	58.88 ± 2.23	32.18 ± 3.13
Ser	10	90.46 ± 7.20	41.80 ± 4.41
Tyr	5	65.15 ± 4.63	46.01 ± 0.74
	10	53.68 ± 4.64	48.68 ± 4.41
Phe	5	23.91± 1.73	18.03 ± 1.70

## Supplementary Materials

The following are available online at https://www.mdpi.com/1996-1944/13/21/4798/s1, Figure S1: Representative pH vs. *t* curves obtained in metastable solutions (MS, *c*(CaCl_2_) = *c*(Na_2_HPO_4_) = 4.198 mmol dm^−3^*, c*(NaCl) = 0.148 mol dm^−3^) with or without added octacalcium phosphate (OCP) seed crystals (*m*OCP = 1 mg) containing 5 and 10 ppm of investigated amino acids: (a) aspartic acid (Asp), (b) tyrosine (Tyr), (c) asparagine (Asn), (d) serine (Ser), (e) lysine (Lys) and (f) phenylalanine (Phe). *t* = 25 °C, pH_initial_ = 7.4, magnetic stirring; Figure S2: Representative pH *vs. t* curves obtained in metastable solutions (MS, *c*(CaCl_2_) = *c*(Na_2_HPO_4_) = 4.198 mmol dm^−3^*, c*(NaCl) = 0.148 mol dm^−3^) with or without added DCPD seed crystals (*m*DCPD = 1 mg) containing 5 and 10 ppm of investigated amino acids: (a) aspartic acid (Asp), (b) tyrosine (Tyr), (c) asparagine (Asn), (d) serine (Ser), (e) lysine (Lys) and (f) phenylalanine (Phe). *t* = 25 °C, pH_initial_ = 7.4, magnetic stirring; Figure S3: FTIR spectra (a,b), PXRD diffractograms (c,d), and SEM micrographs (e, f) of the precipitates formed after 240 min in metastable solutions (*c*(CaCl_2_) = *c*(Na_2_HPO_4_) = 4.198 mmol dm^−3^, *c*(NaCl) = 0.148 mol dm^−3^) containing serine (*γ*(Ser) = 5 ppm, a,c,e) and phenylalanine (*γ*(Phe) = 10 ppm, b,d,f). *t* = 25 °C, pH_initial_ = 7.4, magnetic stirring; Figure S4: FTIR spectra of octacalcium phosphate (OCP) seed crystals and precipitate formed in the control system (CS) and in the presence of different amino acids after aging time corresponding to the commencement of stage III in pH *vs.* time curves in the system *c*(CaCl_2_) = *c*(Na_2_HPO_4_) = 4.198 mmol dm^−3^, *c*(NaCl) = 0.148 mol dm^−3^, *m*(seed OCP) = 1 mg, *γ*(AA) = 10 ppm, except *γ*(Phe) = 5 ppm. *t* = 25 °C, pH_initial_ = 7.4, magnetic stirring. Asp—L-aspartic acid, Tyr—L-tyrosine, Asn—L-asparagine, Lys—L-lysine, Ser—L-serine, Phe—L-phenylalanine; Figure S5: FTIR spectra of octacalcium phosphate (OCP) seed crystals and precipitate formed in the control system (CS) and in the presence of different amino acids after 240 min in the system *c*(CaCl_2_) = *c*(Na_2_HPO_4_) = 4.198 mmol dm^−3^, *c*(NaCl) = 0.148 mol dm^−3^, *m*(seed OCP) = 1 mg, *γ*(AA) = 10 ppm, except *γ*(Phe) = 5 ppm. *t* = 25 °C, pH_initial_ = 7.4, magnetic stirring. Asp—L-aspartic acid, Tyr—L-tyrosine, Asn—L-asparagine, Lys—L-lysine, Ser—L-serine, Phe—L-phenylalanine; Figure S6: FTIR spectra of calcium hydrogenphosphate dihydrate (DCPD) seed crystals and precipitates formed in the control system (CS) and in the presence of different amino acids after 60 min aging time in the system *c*(CaCl_2_) = *c*(Na_2_HPO_4_) = 4.198 mmol dm^−3^, *c*(NaCl) = 0.148 mol dm^−3^, *m*(seed DCPD) = 1 mg, *γ*(AA) = 10 ppm except *γ*(Phe) = 5 ppm *t* = 25 °C, pH_initial_ = 7.4, magnetic stirring. Asp—L-aspartic acid, Tyr—L-tyrosine, Asn—L-asparagine, Lys—L-lysine, Ser—L-serine, Phe—L-phenylalanine; Figure S7: FTIR spectra of calcium hydrogenphosphate dihydrate (DCPD) seed crystals and precipitates formed in the control system (CS) and in the presence of different amino acids after 240 min in the system *c*(CaCl_2_) = *c*(Na_2_HPO_4_) = 4.198 mmol dm^−3^, *c*(NaCl) = 0.148 mol dm^−3^, *m*(seed DCPD) = 1 mg, *γ*(AA) = 10 ppm except *γ*(Phe) = 5 ppm *t* = 25 °C, pH_initial_ = 7.4, magnetic stirring. Asp—L-aspartic acid, Tyr—L-tyrosine, Asn—L-asparagine, Lys—L-lysine, Ser—L-serine, Phe—L-phenylalanine. Table S1: p*K* and pI values of investigated amino acids, *t* = 25 °C. *Asp—L-aspartic acid, Tyr—L-tyrosine, Asn—L-asparagine, Lys—L-lysine, Ser—L-serine, Phe—L-phenylalanine; Table S2: Assignment of reflections in PXRD diffractograms of octacalcium phosphate (OCP) seed crystals and precipitates formed in the control system (CS) and in the presence of different amino acids after aging time corresponding to commencement of stage III in pH *vs.* time curves *c*(CaCl_2_) = *c*(Na_2_HPO_4_) = 4.198 mmol dm^−3^, *c*(NaCl) = 0.148 mol dm^−3^, *m*(seed OCP) = 1 mg, *γ*(AA) = 10 ppm except *γ*(Phe) = 5 ppm *t* = 25 °C, pH_initial_ = 7.4, magnetic stirring. Asp—L-aspartic acid, Tyr—L-tyrosine, Asn—L-asparagine, Lys—L-lysine, Ser—L-serine, Phe—L-phenylalanine; Table S3: Assignment of IR bands in FTIR spectra of octacalcium phosphate (OCP) seed crystals and precipitates formed in the control system (CS) and in the presence of different amino acids after aging time corresponding to commencement of stage III in pH *vs.* time curves in the system *c*(CaCl_2_) = *c*(Na_2_HPO_4_) = 4.198 mmol dm^−3^, *c*(NaCl) = 0.148 mol d^3^, *m*(seed OCP) = 1 mg, *γ*(AA) = 10 ppm, except *γ*(Phe) = 5 ppm. *t* = 25 °C, pH_initial_ = 7.4, magnetic stirring. Asp—L-aspartic acid, TyrL-tyrosine, Asn—L-asparagine, Lys—L-lysine, Ser—L-serine, Phe—L-phenylalanine; Table S4: Intensity of selected phosphate bands and their ratios for octacalcium phosphate (OCP) seed crystals and precipitates formed in the control system (CS) and in the presence of different amino acids in the system *c*(CaCl_2_) = *c*(Na_2_HPO_4_) = 4.198 mmol dm^−3^, *c*(NaCl) = 0.148 mol dm^−3^, *m*(seed OCP) = 1 mg, *γ*(AA) = 10 ppm except *γ*(Phe) = 5 ppm *t* = 25 °C, pH _initial_ = 7.4, magnetic stirring. Asp—L-aspartic acid, Tyr—L-tyrosine, Asn—L-asparagine, Lys—L-lysine, Ser—L-serine, Phe—L-phenylalanine. A1—intensity of HPO_4_ (OH in-plane bending) at around 1285 cm^−1^, A2—*ν*_3_ PO4 stretching at around 1022 cm^−1^, A3—*ν*_4_ HPO_4_ bending at around 557 cm^−1^; Table S5: Assignment of reflections in PXRD diffractograms of octacalcium phosphate (OCP) seed crystals and precipitates formed in the control system (CS) and in the presence of different amino acids after 240 min aging time in the system *c*(CaCl_2_) = *c*(Na_2_HPO_4_) = 4.198 mmol dm^−3^, *c*(NaCl) = 0.148 mol dm^−3^, *m*(seed OCP) = 1 mg, *γ*(AA) = 10 ppm except *γ*(Phe) = 5 ppm *t* = 25 °C, pH_initial_ = 7.4, magnetic stirring. Asp—L-aspartic acid, Tyr—L-tyrosine, Asn—L-asparagine, Lys—L-lysine, Ser—85 L-serine, Phe—L-phenylalanine; Table S6: Assignment of IR bands in FTIR spectra of octacalcium phosphate (OCP) seed crystals (CS) and precipitates formed in the control system and in the presence of different amino acids after 240 min aging time in the system *c*(CaCl_2_) = *c*(Na_2_HPO_4_) = 4.2 mmol dm^−3^, *c*(NaCl) = 0.15 mol dm^−3^, *m*(seed OCP) = 1 mg, *γ*(AA) = 10 ppm except *γ*(Phe) = 5 ppm *t* = 25 °C, pH_initial_ = 7.4, magnetic stirring. Asp—L-aspartic acid, Tyr—L-tyrosine, Asn—L-asparagine, Lys—L-lysine, Ser—L-serine, Phe—L-phenylalanine; Table S7: Assignment of reflections in PXRD diffractograms of calcium hydrogenphosphate dihydrate (DCPD) seed crystals and precipitates formed in the control system (CS) and in the presence of different amino acids after 60 min aging time in the system *c*(CaCl_2_) = *c*(Na_2_HPO_4_) = 4.198 mmol dm^−3^, *c*(NaCl) = 0.148 mol dm^−3^, *m*(seed DCPD) = 1 mg, *γ*(AA) = 10 ppm except *γ*(Phe) = 5 ppm *t* = 25 °C, pH_initial_ = 7.4, magnetic stirring. Asp—L-aspartic acid, Tyr—L-tyrosine, Asn—L-asparagine, Lys—L-lysine, Ser—L-serine, Phe—L-phenylalanine; Table S8: Assignment of IR bands in FTIR spectra of calcium hydrogenphosphate dihydrate (DCPD) seed crystals and precipitates formed in the control system (CS) and in the presence of different amino acids after 60 min aging time in the system *c*(CaCl_2_) = *c*(Na_2_HPO_4_) = 4.198 mmol dm^−3^, *c*(NaCl) = 0.148 mol dm^−3^, *m*(seed DCPD) = 1 mg, *γ*(AA) = 10 ppm except *γ*(Phe) = 5 ppm *t* = 25 °C, pH_initial_ = 7.4, magnetic stirring. Asp—L-aspartic acid, Tyr—L-tyrosine, Asn—L-asparagine, Lys—L-lysine, Ser—L-serine, Phe—L-phenylalanine; Table S9: Intensity of selected phosphate bands and their ratios for calcium hydrogenphosphate dihydrate (DCPD) seed crystals and precipitates formed in the control system (CS) and in the presence of different amino acids in the system *c*(CaCl_2_) = *c*(Na_2_HPO_4_) = 4.198 mmol dm^−3^, *c*(NaCl) = 0.148 mol dm^−3^, *m*(seed DCPD) = 1 mg, *γ*(AA) = 10 ppm except *γ*(Phe) = 5 ppm *t* = 25 °C, pH_initial_ = 7.4, magnetic stirring. Asp—L-aspartic acid, Tyr—L-tyrosine, Asn—L-asparagine, Lys—L-lysine, Ser—L-serine, Phe—L-phenylalanine. A1—intensity of P-O(H) stretching at around 872 cm^−1^, A2—*ν*_4c_ PO bending (O-P-O) at around 560 cm^−1^, A3—*ν*_2_ PO bending (O-P-O) at around 525 236 cm^−1^; Table S10: Assignment of reflections in PXRD diffractograms of calcium hydrogenphosphate dihydrate (DCPD) seed crystals and precipitates formed in the control system (CS) and in the presence of different amino acids after 240 min aging time in the system *c*(CaCl_2_) = *c*(Na_2_HPO_4_) = 4.198 mmol dm^−3^, *c*(NaCl) = 0.148 mol dm^−3^, *m*(seed DCPD) = 1 mg, *γ*(AA) = 10 ppm except *γ*(Phe) = 5 ppm *t* = 25 °C, pH_initial_ = 7.4, magnetic stirring. Asp—L-aspartic acid, Tyr—L-tyrosine, Asn—L-asparagine, Lys—L-lysine, Ser—L-serine, Phe—L-phenylalanine; Table S11: Assignment of IR bands in FTIR spectra of calcium hydrogenphosphate dihydrate (DCPD) seed crystals and precipitates formed in the control system (CS) and in the presence of different amino acids after 240 min aging time in the system *c*(CaCl_2_) = *c*(Na_2_HPO_4_) = 4.198 mmol dm^−3^, *c*(NaCl) = 0.148 mol dm^−3^, *m*(seed DCPD) = 1 mg, *γ*(AA) = 10 ppm except *γ*(Phe) = 5 ppm *t* = 25 °C, pH_initial_ *l* = 7.4, magnetic stirring. Asp—L-aspartic acid, Tyr—L-tyrosine, Asn—L-asparagine, Lys—L-lysine, Ser—L-serine, Phe—L-phenylalanine.

## References

[1] Lowenstam H.A., Weiner S. (1989). On Biomineralization.

[2] Mann S. (2001). Biomineralization: Principles and Concepts in Bioinorganic Materials Chemistry.

[3] LeGeros R.Z. (2008). Calcium Phosphate-Based Osteoinductive Materials. Chem. Rev..

[4] Crichton R.R. (2008). Biological Inorganic Chemistry.

[5] Weiner S., Dove P.M. (2003). An Overview of Biomineralization Processes and the Problem of the Vital Effect. Rev. Mineral. Geochem..

[6] Addadi L., Joester D., Nudelman F., Weiner S. (2006). Mollusk shell formation: A source of new concepts for understanding biomineralization processes. Chem. Eur. J..

[7] Dickerson M.B., Sandhage K.H., Naik R.R. (2008). Protein- and peptide-directed syntheses of inorganic materials. Chem. Rev..

[8] Falini G., Fermani S. (2013). The strategic role of adsorption phenomena in biomineralization. Cryst. Res. Technol..

[9] Dorozhkin S.V. (2012). Calcium Orthophosphates: Applications in Nature, Biology, and Medicine.

[10] Dorozhkin S.V., Epple M. (2002). Biological and medical significance of calcium phosphates. Angew. Chem. Int. Ed..

[11] Tung M.S., Amjad Z. (1998). Calcium Phosphates: Structure, Composition, Solubility, and Stability. Calcium Phosphates in Biological and Industrial Systems.

[12] Zhang Z.L., Chen X.R., Bian S., Huang J., Zhang T.L., Wang K. (2014). Identification of dicalcium phosphate dihydrate deposited during osteoblast mineralization in vitro. J. Inorg. Biochem..

[13] Graham S., Brown P.W. (1996). Reactions of octacalcium phosphate to form hydroxyapatite. J. Cryst. Growth.

[14] Zhang J., Nancollas G.H. (1992). Kinetics and mechanisms of octacalcium phosphate dissolution at 37 °C. J. Phys. Chem..

[15] Arellano-Jiménez M.J., García-García R., Reyes-Gasga J. (2009). Synthesis and hydrolysis of octacalcium phosphate and its characterization by electron microscopy and X-ray diffraction. J. Phys. Chem. Solids.

[16] Brown P.W., Fulmer M. (1991). Kinetics of Hydroxyapatite Formation at Low Temperature. J. Am. Ceram. Soc..

[17] Bodier-Houllé P., Steuer P., Meyer J.M., Bigeard L., Cuisinier F.J.G. (2000). High-resolution electron-microscopic study of the relationship between human enamel and dentin crystals at the dentinoenamel junction. Cell Tissue Res..

[18] Xin R., Leng Y., Wang N. (2006). In situ TEM examinations of octacalcium phosphate to hydroxyapatite transformation. J. Cryst. Growth.

[19] Chu X., Jiang W., Zhang Z., Yan Y., Pan H., Xu X., Tang R. (2011). Unique Roles of Acidic Amino Acids in Phase Transformation of Calcium Phosphates. J. Phys. Chem. B.

[20] Suzuki M., Saruwatari K., Kogure T., Yamamoto Y., Nishimura T., Kato T., Nagasawa H. (2009). An Acidic Matrix Protein, Pif, Is a Key Macromolecule for Nacre Formation. Science.

[21] Jahromi M.T., Yao G., Cerruti M. (2012). The importance of amino acid interactions in the crystallization of hydroxyapatite. J. R. Soc. Interface.

[22] Tavafoghi M., Cerruti M. (2016). The role of amino acids in hydroxyapatite mineralization. J. R. Soc. Interface.

[23] Rubini K., Boanini E., Bigi A. (2019). Role of aspartic and polyaspartic acid on the synthesis and hydrolysis of brushite. J. Funct. Biomater..

[24] Gonzalez-McQuire R., Chane-Ching J.Y., Vignaud E., Lebugle A., Mann S. (2004). Synthesis and characterization of amino acid-functionalized hydroxyapatite nanorods. J. Mater. Chem..

[25] Boanini E., Torricelli P., Gazanno M., Giardino R., Bigi A. (2006). Nanocomposites of hydroxyapatite with aspartic acid and glutamic acid and their interaction with osteoblast-like cells. Biomaterials.

[26] Palazzo B., Walsh D., Iafisco M., Foresti E., Bertinetti L., Martra G., Bianchi C.L., Cappelletti G., Roveri N. (2009). Amino acid synergetic effect on structure, morphology and surface properties of biomimetic apatite nanocrystals. Acta Biomater..

[27] Jack K.S., Vizcarra T.G., Trau M. (2007). Characterization and Surface Properties of Amino-Acid-Modified Carbonate-Containing Hydroxyapatite Particles. Langmuir.

[28] Yang X., Xie B., Wang L., Qin Y., Henneman Z.J., Nancollas G.H. (2011). Influence of magnesium ions and amino acids on the nucleation and growth of hydroxyapatite. Cryst. Eng. Comm..

[29] Koutsoupolos S., Dalas E. (2001). Hydroxyapatite Crystallization in the Presence of Amino Acids with Uncharged Polar Side Groups:  Glycine, Cysteine, Cystine, and Glutamine. Langmuir.

[30] Tsai T.W.T., Chen W.Y., Tseng Y.H., Chan J.C.C. (2011). Phase transformation of calcium phosphates in the presence of glutamic acid. Can. J. Chem..

[31] Sikirić M., Babić-Ivančić V., Milat O., Sarig S., Füredi-Milhofer H. (2000). Factors Influencing Additive Interactions with Calcium Hydrogenphosphate Dihydrate Crystals. Langmuir.

[32] Gilman H., Hukins D.W.L. (1994). Seeded growth of hydroxyapatite in the presence of dissolved albumin. J. Inorg. Biochem..

[33] Weiner S., Wagner H.D. (1998). The Material Bone: Structure-Mechanical Function Relations. Ann. Rev. Mater. Sci..

[34] Abou Neel E., Aljabo A., Strange A., Ibrahim S., Coathup M., Young A., Bozec L., Mudera V. (2016). Demineralization & remineralization dynamics in teeth and bone. Inter. J. Nanomed..

[35] Elliott J.C. (1994). Structure and chemistry of the apatites and other calcium orthophosphates. Stud. Org. Chem..

[36] Furedi-Milhofer H., Moradian-Oldak J., Weiner S., Veis A., Mintz K.P., Addadf L. (1994). Interactions of matrix proteins from mineralized tissues with octacalcium phosphate. Connect. Tissue Res..

[37] Rey C. (1998). Calcium Phosphates for Medical Applications. Calcium Phosphates in Biological and Industrial Systems.

[38] Hanein D., Geiger B., Addadi L. (1993). Fibronectin Adsorption to Surfaces of Hydrated Crystals. An Analysis of the Importance of Bound Water in Protein-Substrate Interactions. Langmuir.

[39] Abbona F., Christensson F., Angela M.F., Madsen H.E.L. (1993). Crystal habit and growth conditions of brushite, CaHPO_4_ 2H_2_O. J. Cryst. Growth.

[40] Markovic M. (2001). Octacalcium Phosphate.

[41] Monma H., Moriyoshi Y. (1990). Zeolitic dehydration-rehydration of adipate-intercalated octacalcium phosphate. J. Mater. Sci..

[42] Nancollas G.H., Wu W. (2000). Biomineralization mechanisms: A kinetics and interfacial energy approach. J. Cryst. Growth.

[43] Brown W.E., Mathew M., Chow L.C. (1984). Adsorption on and Surface Chemistry of Hydroxyapatite.

[44] Bigi A., Bracci B., Panzavolta S., Iliescu M., Plouet-Richard M., Werckmann J., Cam D. (2004). Morphological and Structural Modifications of Octacalcium Phosphate Induced by Poly-L-Aspartate. Cryst. Growth Des..

[45] LeGeros R.Z. (1985). Preparation of octacalcium phosphate (OCP): A direct fast method. Calcif. Tissue Int..

[46] Buljan Meić I., Kontrec J., Domazet Jurašin D., Njegić Džakula B., Štajner L., Lyons D.M., Dutour Sikirić M., Kralj D. (2017). Comparative Study of Calcium Carbonates and Calcium Phosphates Precipitation in Model Systems Mimicking the Inorganic Environment for Biomineralization. Cryst. Growth Des..

[47] Wang L., Nancollas G.H. (2009). Pathways to biomineralization and biodemineralization of calcium phosphates: The thermodynamic and kinetic controls. Dalton Trans..

[48] Selmani A., Coha I., Magdić K., Čolović B., Jokanović V., Šegota S., Gajović S., Gajović A., Jurašin D., Dutour Sikirić M. (2015). Multiscale study of the influence of cationic surfactants on amorphous calcium phosphate precipitation. Cryst. Eng. Comm..

[49] Ofir P.B.-Y., Govrin-Lippman R., Garti N., Füredi-Milhofer H. (2004). The Influence of Polyelectrolytes on the Formation and Phase Transformation of Amorphous Calcium Phosphate. Cryst. Growth Des..

[50] Li S., Wang L. (2012). Phosphorylated osteopontin peptides inhibit crystallization by resisting the aggregation of calcium phosphate nanoparticles. Cryst. Eng. Comm..

[51] Jahromi T.M., Cerruti M. (2015). Amino acid/ion aggregate formation and their role in hydroxyapatite precipitation. Cryst. Growth Des..

[52] Suzuki O. (2010). Octacalcium phosphate: Osteoconductivity and crystal chemistry. Acta Biomater..

[53] Koutsopoulos S. (2002). Synthesis and characterization of hydroxyapatite crystals: A review study on the analytical methods. J. Biomed. Mater. Res..

[54] Fowler B.O., Marković M., Brown W.E. (1993). Octacalcium Phosphate. 3. Infrared and Raman Vibrational Spectra. Chem. Mater..

[55] Nancollas G.H., Wefel J.S. (1976). Seeded Growth of Calcium Phosphates: Effect of Different Calcium Phosphate Seed Material. J. Dent. Res..

[56] Bigi A., Boanini E., Falini G., Panzavolta S., Roveri N. (2000). Effect of sodium polyacrylate on the hydrolysis of octacalcium phosphate. J. Inorg. Biochem..

[57] Bigi A., Boanini E., Bracci B., Falini G., Rubini K. (2003). Interaction of acidic poly-amino acids with octacalcium phosphate. J. Inorg. Biochem..

[58] Dorozhkin S. (2010). V Amorphous calcium (ortho)phosphates. Acta Biomater..

[59] Karampas I.A., Kontoyannis C.G. (2013). Characterization of calcium phosphates mixtures. Vib. Spectroscop..

[60] Xu J., Butler I.S., Gilson D.F.R. (1999). FT-Raman and high-pressure infrared spectroscopic studies of dicalcium phosphate dihydrate (CaHPO_4_·2H_2_O) and anhydrous dicalcium phosphate (CaHPO_4_). Spectochim. Acta Part A.

[61] Obadia L., Rouillon T., Bujoli B., Daculsi G., Bouler J.M. (2007). Calcium-deficient apatite synthesized by ammonia hydrolysis of dicalcium phosphate dihydrate: Influence of temperature, time, and pressure. J. Biomed. Mater. Res. Part B.

[62] Tas A.C., Bhaduri S.B. (2004). Chemical Processing of CaHPO_4_·2H_2_O: Its Conversion to Hydroxyapatite. J. Am. Ceram. Soc..

[63] Štulajterová R., Medvecký Ľ. (2008). Effect of calcium ions on transformation brushite to hydroxyapatite in aqueous solutions. Colloids Surf. A.

