# Supplementary material for: The Influence of Different Classes of Amino Acids on Calcium Phosphates Seeded Growth
Source: Materials (Basel). 2020 Oct 27;13(21):4798. doi: 10.3390/ma13214798 (PMC7662258; doi:10.3390/ma13214798)
Supplement: Supplementary file 1 [file materials-13-04798-s001.pdf]

Supplementary Material

The Influence of Different Classes of Amino Acids on Calcium Phosphates Seeded Growth

Table S1. p*K* and pI values of investigated amino acids, *t* = 25 °C. *Asp—L–aspartic acid, Tyr—L–tyrosine, Asn—L–asparagine, Lys—L–lysine, Ser—L–serine, Phe—L–phenylalanine.

Amino Acid	pK_1 (α-COOH Group)	pK_2 (α-NH_3^+ Group)	pK_3 (Side Chain)	pI
**Asp**	2.0	10.0	3.9	2.77
**Lys**	2.2	9.2	10.8	9.74
**Asn**	2.0	8.8	-	5.41
**Ser**	2.1	9.2	-	5.68
**Tyr**	2.2	9.1	10.9	5.66
**Phe**	1.8	9.1	-	5.48

* L. Stryer, Biochemistry, 4th edition, W.H. Freeman and Company, New York, 1995.

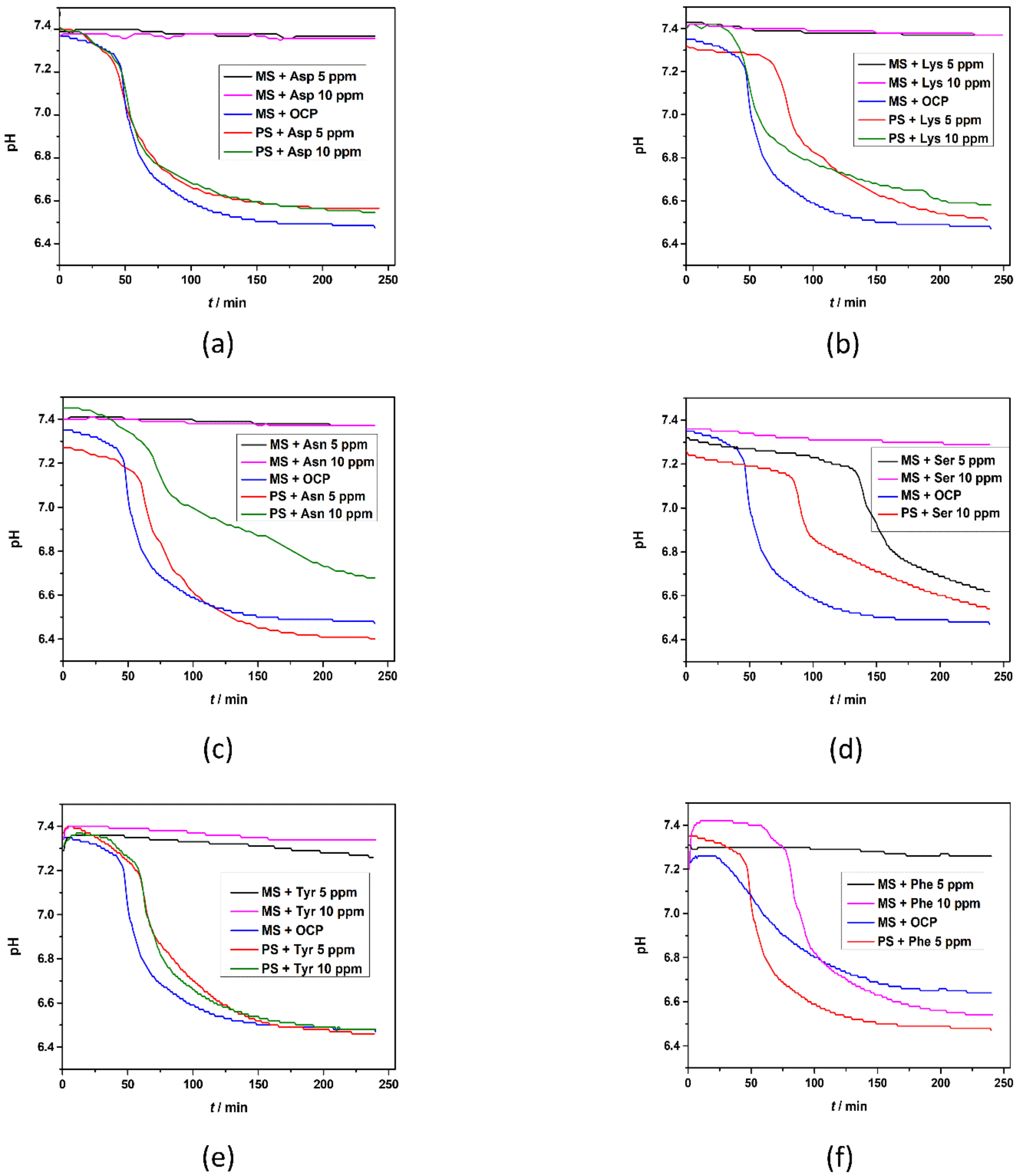

Figure S1. Representative pH *vs. t* curves obtained in metastable solutions (MS, $c(CaCl_2) = c(Na_2HPO_4)$ = 4.198 mmol dm^{-3}, c(NaCl) = 0.148 mol dm^{-3}) with or without added octacalcium phosphate (OCP) seed crystals (mOCP = 1 mg) containing 5 and 10 ppm of investigated amino acids: (**a**) aspartic acid (Asp), (**b**) tyrosine (Tyr), (c) asparagine (Asn), (**d**) serine (Ser), (**e**) lysine (Lys) and (**f**) phenylalanine (Phe). t = 25 °C, $pH_{initial}$ = 7.4, magnetic stirring.

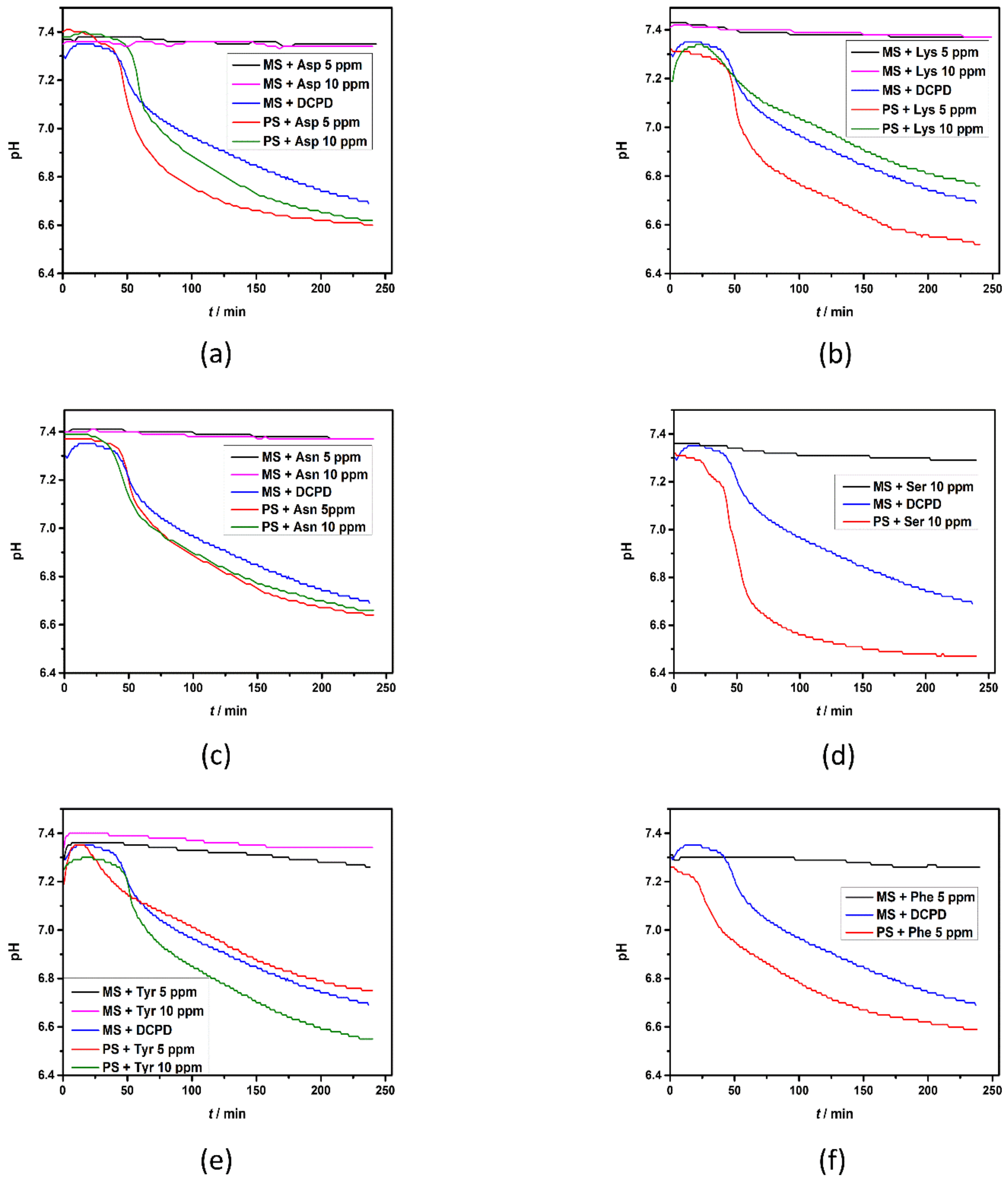

Figure S2. Representative pH *vs.* t curves obtained in metastable solutions (MS, $c(CaCl_2) = c(Na_2HPO_4) = 4.198$ mmol dm^{-3}, $c(NaCl) = 0.148$ mol dm^{-3}) with or without added DCPD seed crystals (mDCPD = 1 mg) containing 5 and 10 ppm of investigated amino acids: (**a**) aspartic acid (Asp), (**b**) tyrosine (Tyr), (**c**) asparagine (Asn), (**d**) serine (Ser), (**e**) lysine (Lys) and (**f**) phenylalanine (Phe). t = 25 °C, $pH_{initial}$ = 7.4, magnetic stirring.

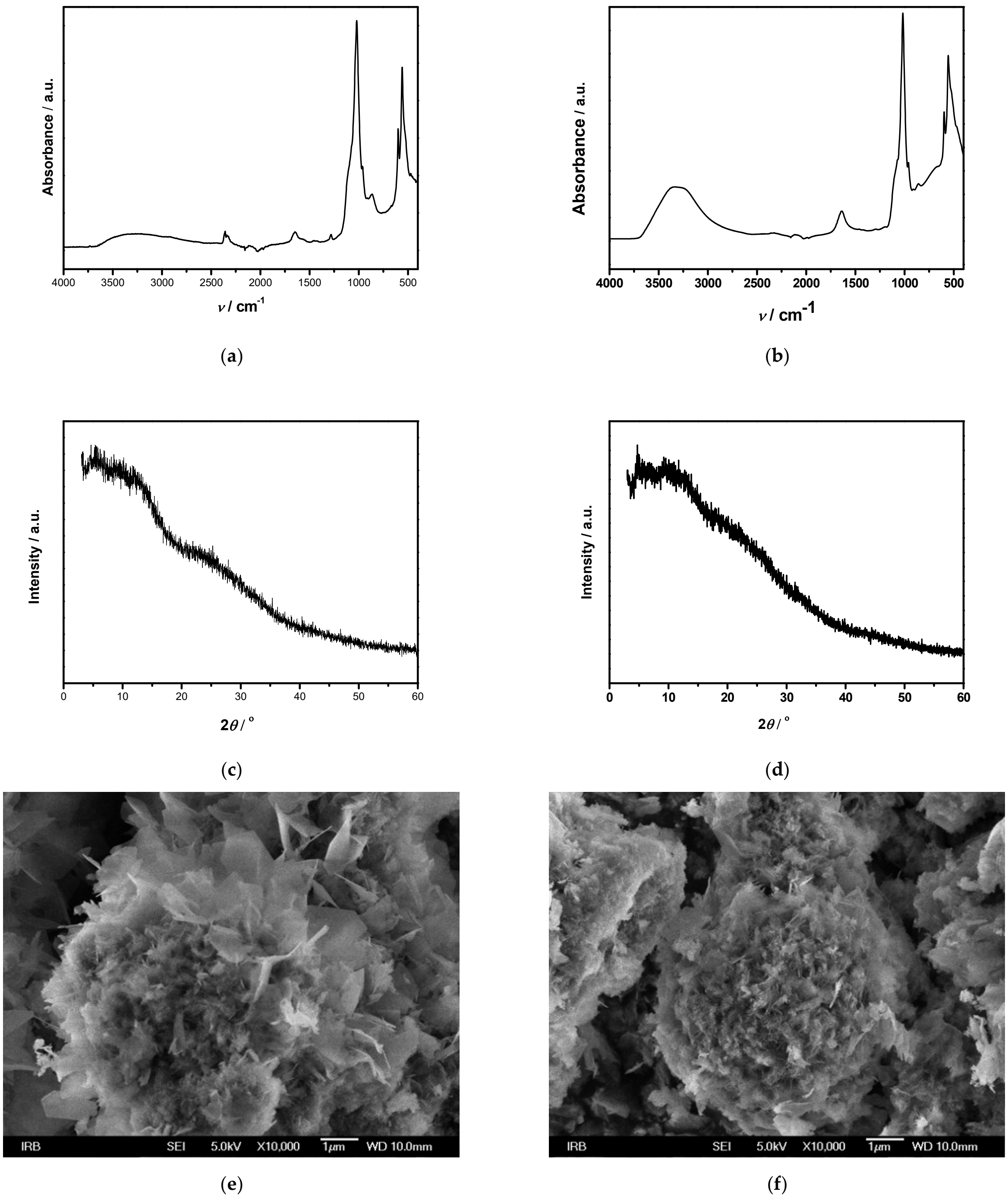

Figure S3. FTIR spectra (**a**,**b**), PXRD diffractograms (**c**,**d**), and SEM micrographs (**e**,**f**) of the precipitates formed after 240 min in metastable solutions ($c(CaCl_2) = c(Na_2HPO_4) = 4.198$ mmol dm^{-3}, $c(NaCl) = 0.148$ mol dm^{-3}) containing serine (γ(Ser) = 5 ppm, a,c,e) and phenylalanine (γ(Phe) = 10 ppm, b,d,f). $t = 25$ °C, $pH_{initial} = 7.4$, magnetic stirring.

Table S2. Assignment of reflections in PXRD diffractograms of octacalcium phosphate (OCP) seed crystals and precipitates formed in the control system (CS) and in the presence of different amino acids after aging time corresponding to commencement of stage III in pH *vs.* time curves $c(CaCl_2) = c(Na_2HPO_4) = 4.198$ mmol dm^{-3}, $c(NaCl) = 0.148$ mol dm^{-3}, m(seed OCP) = 1 mg, γ(AA) = 10 ppm except

γ(Phe) = 5 ppm t = 25 °C, $pH_{initial}$ = 7.4, magnetic stirring. Asp—L–aspartic acid, Tyr—L–tyrosine, Asn—L–asparagine, Lys—L–lysine, Ser—L–serine, Phe—L–phenylalanine.

**2θ/°**								**hkl**
**Seed**	**CS**	**Asp**	**Lys**	**Asn**	**Ser**	**Tyr**	**Phe**	
4.86	4.73	4.72			4.96			(100)
9.42			9.45	9.55	9.61		9.61	$(\bar{1}10)$
9.89								(010)
16.14								$(1\bar{1}\bar{1})$
							22.61	$(1\bar{2}1)$
22.92								$(2\bar{2}1)$
23.88								(311)
24.52	24.36							$(3\bar{2}1)$
25.66								$(\bar{4}21)$
	26.16	25.95		25.94	26.03	26.01		CaDHA
26.25								$(\bar{1}02)$
27.38								$(\bar{2}\bar{2}1)$
28.01								$(\bar{2}12)$
29.35								$(\bar{4}30)$
30.49								$(6\bar{1}1)$
31.68	31.61	31.50	31.61	31.71	31.61	31.74		$(\bar{4}02)$
		32.10						CaDHA
32.42								$(4\bar{1}2)$
33.77			33.60					$(\bar{7}11)$
34.14								(131)
35.23								$(5\bar{1}2)$
36.44								(701)
	36.75							$(\bar{7}30)$
40.78	40.86							$(\bar{6}40)$
43.06								(612)
		46.70			46.70	46.81		CaDHA
		49.58						CaDHA
		53.15						CaDHA

Assignments made according to: a JCPDS No. 074-1301; b Koutsopoulos, S. Synthesis and Characterization of Hydroxyapatite Crystals: A Review Study on the Analytical Methods. *J. Biomed.Mater. Res*. 2002, 62 (4), 600–612; c Karampas, L.A., Kontoyannis, C.G., Characterization of calcium phosphates mixtures. *Vib. Spec*. 2013, 64, 126–133.

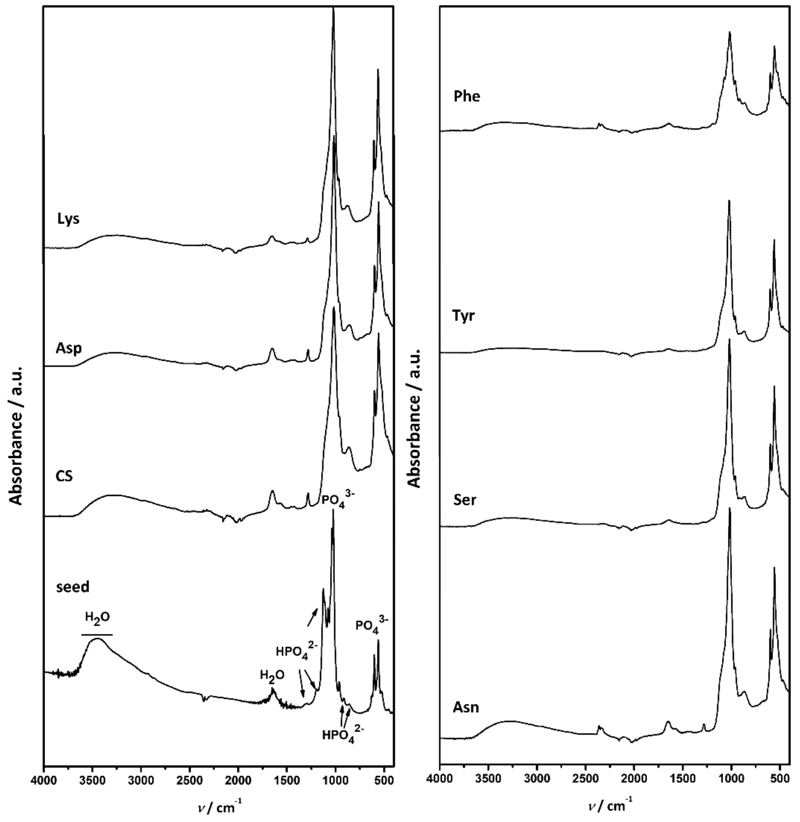

Figure S4. FTIR spectra of octacalcium phosphate (OCP) seed crystals and precipitate formed in the control system (CS) and in the presence of different amino acids after aging time corresponding to the commencement of stage III in pH *vs.* time curves in the system $c(CaCl_2) = c(Na_2HPO_4) = 4.198$ mmol dm^{-3}, $c(NaCl) = 0.148$ mol dm^{-3}, m(seed OCP) = 1 mg, γ(AA) = 10 ppm, except γ(Phe) = 5 ppm. t = 25 °C, $pH_{initial} = 7.4$, magnetic stirring. Asp—L−aspartic acid, Tyr—L−tyrosine, Asn—L−asparagine, Lys—L−lysine, Ser—L−serine, Phe—L−phenylalanine.

Table S3. Assignment of IR bands in FTIR spectra of octacalcium phosphate (OCP) seed crystals and precipitates formed in the control system (CS) and in the presence of different amino acids after aging time corresponding to commencement of stage III in pH *vs.* time curves in the system $c(CaCl_2) = c(Na_2HPO_4) = 4.198$ mmol dm^{-3}, c(NaCl) = 0.148 mol d^3, m(seed OCP) = 1 mg, γ(AA) = 10 ppm, except γ(Phe) = 5 ppm. t = 25 °C, $pH_{initial}$ = 7.4, magnetic stirring. Asp—L−aspartic acid, TyrL−tyrosine, Asn—L−asparagine, Lys—L−lysine, Ser—L−serine, Phe—L−phenylalanine.

Wavenumber/cm^{-1}								Band Assignment
**Seed**	**CS**	**Asp**	**Lys**	**Asn**	**Ser**	**Tyr**	**Phe**	
3619–2579	3670–2540	3647–2551	3664–2542	3682–2579	3660–2565	3663–2579	3670–2575	water vibration[a,b]
				2348			2334	HPO_4 (OH) stretching[b]
1636	1648	1650	1651	1650	1649	1647	1642	water vibration[a,b]
1293	1282	1282	1286	1284	1286	1282	1288	HPO_4 (OH in-plane bending)[b]
1192					1194		1194	HPO_4 (OH in-plane bendimg)[b]
1131								ν_3 HPO_4 stretching[b]
1073							1075	ν_3 HPO_4, ν_3 PO_4 stretching[b]
1028	1018	1014	1018	1018	1019	1018	1018	ν_3 PO_4 stretching[b]
957	959	955	963	963	963	959	964	ν_1 PO_4 stretching[b]
915					913	913	915	HPO_4 (P-OH) stretching[b]
856	864	854	864	865	865	860	863	HPO_4 (P-OH) stretching[b]
602	596	601	596	601	601	601	602	ν_4 PO_4 bending[b]
560	554	553	562	555	560	560	560	$\nu_4 HPO_4$ bending[b]
525								$\nu_4 HPO_4$ bending[b]
453	463	461	461	466	466	466	466	$\nu_2 PO_4$ bending[b]
						448	447	H_2O libration[b]

[a] Koutsopoulos, S. Synthesis and Characterization of Hydroxyapatite Crystals: A Review Study on the Analytical Methods. *J. Biomed.Mater. Res.* **2002**, *62* (4), 600–612; [b] Fowler, B. O.; Markovic, M.; Brown, W. E. Octacalcium Phosphate. 3. Infrared and Raman Vibrational Spectra. *Chem. Mater.* **1993**, *5* (10), 1417–1423.

Table S4. Intensity of selected phosphate bands and their ratios for octacalcium phosphate (OCP) seed crystals and precipitates formed in the control system (CS) and in the presence of different amino acids in the system $c(CaCl_2) = c(Na_2HPO_4)$ = 4.198 mmol dm^{-3}, c(NaCl) = 0.148 mol dm^{-3}, m(seed OCP) = 1 mg, γ(AA) = 10 ppm except γ(Phe) = 5 ppm t = 25 °C, pH$_{initial}$ = 7.4, magnetic stirring. Asp—L–aspartic acid, Tyr—L–tyrosine, Asn—L–asparagine, Lys—L–lysine, Ser—L–serine, Phe—L–phenylalanine. A1—intensity of HPO_4 (OH in-plane bending) at around 1285 cm^{-1}, A2—ν_3 PO4 stretching at around 1022 cm^{-1}, A3—ν_4 HPO_4 bending at around 557 cm^{-1}.

**Aging Time Corresponding to Commencement of Stage III**						
	**A1**	**A2**	**A3**	**A1:A2**	**A1:A3**	**A2:A3**
Seed	0.037	0.442	0.159	0.08	0.23	2.78
CS	0.008	0.101	0.007	0.08	1.14	14.43
Asp	0.022	0.406	0.247	0.05	0.09	1.64
Lys	0.006	0.303	0.194	0.02	0.03	1.56
Asn	0.023	0.313	0.232	0.07	0.10	1.35
Ser	0.009	0.286	0.215	0.03	0.04	1.33
Tyr	0.008	0.188	0.148	0.04	0.05	1.27
Phe	0.007	0.128	0.110	0.05	0.06	1.16
**240 min Aging Time**						
CS	0.023	0.354	0.223	0.06	0.10	1.59
Asp	0.022	0.290	0.176	0.08	0.13	1.65
Lys	0.002	0.450	0.266	0.00	0.01	1.69
Asn	0.016	0.437	0.251	0.04	0.06	1.74
Ser	0.004	0.233	0.159	0.02	0.03	1.47
Tyr	-	0.283	0.189	-	-	1.50
Phe	0.003	0.339	0.224	0.01	0.01	1.51

Table S5. Assignment of reflections in PXRD diffractograms of octacalcium phosphate (OCP) seed crystals and precipitates formed in the control system (CS) and in the presence of different amino acids after 240 min aging time in the system $c(CaCl_2) = c(Na_2HPO_4)$ = 4.198 mmol dm^{-3}, c(NaCl) = 0.148 mol dm^{-3}, m(seed OCP) = 1 mg, γ(AA) = 10 ppm except γ(Phe) = 5 ppm t = 25 °C, pH$_{initial}$ = 7.4, magnetic stirring. Asp—L–aspartic acid, Tyr—L–tyrosine, Asn—L–asparagine, Lys—L–lysine, Ser—L–serine, Phe—L–phenylalanine.

**2θ/°**								**hkl**
**Seed**	**CS**	**Asp**	**Lys**	**Asn**	**Ser**	**Tyr**	**Phe**	
4.86	4.65	4.77	4.64	4.61				(100)
9.42	9.61		9.56		9.54		9.39	($\bar{1}$10)
9.89								(010)
16.14								(1$\bar{1}$1)
22.92	22.83							(2$\bar{2}$1)
23.88								(311)
24.52								(3$\bar{2}$1)
25.66								($\bar{4}$21)
	25.95	25.95	25.97	25.92	25.89	25.88	25.94	CaDHA
26.25								($\bar{1}$02)
27.38								($\bar{2}\bar{2}$1)
			27.87					(012)
27.99								($\bar{2}$12)
29.35			29.20					($\bar{4}$30)
30.49								(6$\bar{1}$1)
31.68								($\bar{4}$02)
	31.57	31.51	31.51	31.66	31.74	31.62	31.55	CaDHA
32.42								(4$\bar{1}$2)
33.77								($\bar{7}$11)
34.14								(131)
35.23								(5$\bar{1}$2)
36.44								(701)
				39.55	39.55			(040)
40.78								($\bar{6}$40)
		41.96						CaDHA
43.11								(612)
	46.70	46.85	46.70	46.55		46.63		CaDHA

	49.65	49.65	49.34	49.68	49.74	49.58	49.56	CaDHA
	53.33	53.42	53.42	53.26	53.39	53.93		CaDHA

Assignments made according to: [a] JCPDS No. 074-1301; [b] Koutsopoulos, S. Synthesis and Characterization of Hydroxyapatite Crystals: A Review Study on the Analytical Methods. *J. Biomed.Mater. Res*. 2002, 62 (4), 600–612; [c] Karampas, L.A., Kontoyannis, C.G., Characterization of calcium phosphates mixtures. *Vib. Spec.* 2013, 64, 126–133.

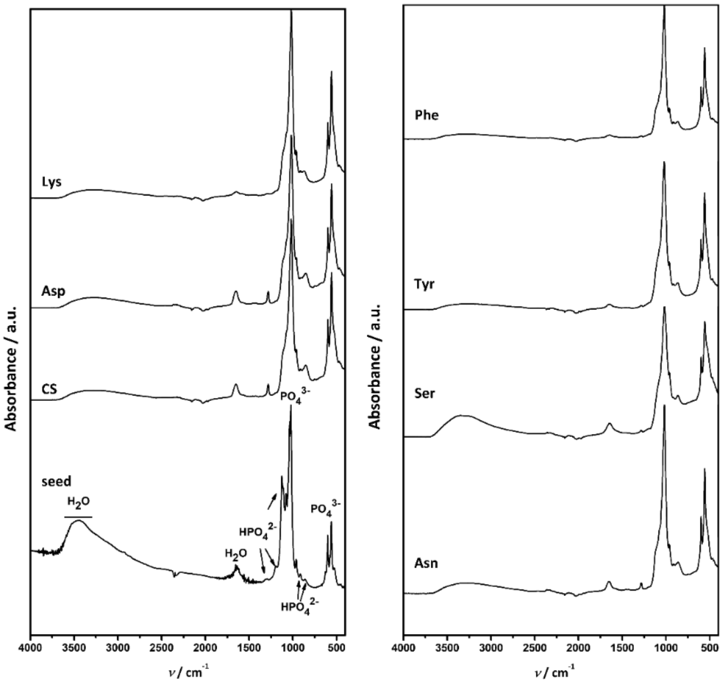

Figure S5. FTIR spectra of octacalcium phosphate (OCP) seed crystals and precipitate formed in the control system (CS) and in the presence of different amino acids after 240 min in the system $c(CaCl_2)$ = $c(Na_2HPO_4)$ = 4.198 mmol dm^{-3}, c(NaCl) = 0.148 mol dm^{-3}, m(seed OCP) = 1 mg, γ(AA) = 10 ppm, except γ(Phe) = 5 ppm. t = 25 °C, $pH_{initial}$ = 7.4, magnetic stirring. Asp—L–aspartic acid, Tyr—L–tyrosine, Asn—L–asparagine, Lys—L–lysine, Ser—L–serine, Phe—L–phenylalanine.

Table S6. Assignment of IR bands in FTIR spectra of octacalcium phosphate (OCP) seed crystals and precipitates formed in the control system (CS) and in the presence of different amino acids after aging time corresponding to commencement of stage III in pH *vs.* time curves in the system $c(CaCl_2) = c(Na_2HPO_4) = 4.198$ mmol dm^{-3}, c(NaCl) = 0.148 mol d^3, m(seed OCP) = 1 mg, γ(AA) = 10 ppm, except γ(Phe) = 5 ppm. t = 25 °C, $pH_{initial}$ = 7.4, magnetic stirring. Asp—L−aspartic acid, TyrL−tyrosine, Asn—L−asparagine, Lys—L−lysine, Ser—L−serine, Phe—L−phenylalanine.

Wavenumber/cm⁻¹								Band Assignment
**Seed**	**CS**	**Asp**	**Lys**	**Asn**	**Ser**	**Tyr**	**Phe**	
3619–2579	3670–2540	3647–2551	3664–2542	3682–2579	3660–2565	3663–2579	3670–2575	water vibration[a,b]
				2348			2334	HPO_4 (OH) stretching[b]
1636	1648	1650	1651	1650	1649	1647	1642	water vibration[a,b]
1293	1282	1282	1286	1284	1286	1282	1288	HPO_4 (OH in-plane bending)[b]
1192					1194		1194	HPO_4 (OH in-plane bendimg)[b]
1131								ν_3 HPO_4 stretching[b]
1073							1075	ν_3 HPO_4, ν_3 PO_4 stretching[b]
1028	1018	1014	1018	1018	1019	1018	1018	ν_3 PO_4 stretching[b]
957	959	955	963	963	963	959	964	ν_1 PO_4 stretching[b]
915					913	913	915	HPO_4 (P-OH) stretching[b]
856	864	854	864	865	865	860	863	HPO_4 (P-OH) stretching[b]
602	596	601	596	601	601	601	602	ν_4 PO_4 bending[b]
560	554	553	562	555	560	560	560	$\nu_4 HPO_4$ bending[b]
525								$\nu_4 HPO_4$ bending[b]
453	463	461	461	466	466	466	466	$\nu_2 PO_4$ bending[b]
						448	447	H_2O libration[b]

[a] Koutsopoulos, S. Synthesis and Characterization of Hydroxyapatite Crystals: A Review Study on the Analytical Methods. *J. Biomed.Mater. Res.* **2002**, *62* (4), 600–612; [b] Fowler, B. O.; Markovic, M.; Brown, W. E. Octacalcium Phosphate. 3. Infrared and Raman Vibrational Spectra. *Chem. Mater.* **1993**, *5* (10), 1417–1423.

Table S7. Assignment of reflections in PXRD diffractograms of calcium hydrogenphosphate dihydrate (DCPD) seed crystals and precipitates formed in the control system (CS) and in the presence of different amino acids after 60 min aging time in the system $c(CaCl_2) = c(Na_2HPO_4)$ = 4.198 mmol dm^{-3}, c(NaCl) = 0.148 mol dm^{-3}, m(seed DCPD) = 1 mg, γ(AA) = 10 ppm except γ(Phe) = 5 ppm t = 25 °C, $pH_{initial}$ = 7.4, magnetic stirring. Asp—L–aspartic acid, Tyr—L–tyrosine, Asn—L–asparagine, Lys—L–lysine, Ser—L–serine, Phe—L–phenylalanine.

2θ/°								hkl
**Seed**	**CS**	**Asp**	**Lys**	**Asn**	**Ser**	**Tyr**	**Phe**	
11.58	11.61	11.60	11.51	11.88	11.53	11.70	11.51	(020)
20.91	20.89	20.97	20.89	20.94			20.87	(021)
23.31		23.36						(040)
							23.64	
		25.87						CaDHA
25.98								($\bar{1}$31)
29.22	29.29	29.29	29.29	29.30			29.27	(041)
30.52	30.69	30.52	30.51				30.42	($\bar{2}$21)
31.84								(200)
		31.62	31.62					CaDHA
34.08	34.21	34.15	34.21	34.30			34.11	($\bar{2}$20)
36.96	37.06	36.97	36.93				36.95	(022)
39.69								(220)
41.63		41.65	41.64				41.64	(151)
	41.73				41.80			
42.05								($\bar{2}$42)
42.97								($\bar{1}$52)
			43.06					
43.29		43.24						($\bar{3}$11)
44.71								(170)
				44.93	45.00		45.06	
45.19			45.15					($\bar{1}$71)
45.96	45.78							(112)
47.99								(080)
48.46	48.54	48.41	48.50		48.54			($\bar{2}$60)
50.13	50.12						50.15	(241)
		50.28	50.21					
50.83								(062)
51.42								(081)
53.53	53.48	53.39	53.46				53.51	(18$\bar{1}$)
55.27								(25$\bar{3}$)
56.52								(181)
58.79								(082)
59.54			59.56					(20$\bar{4}$)

Assignment made according to: JCPDS card No. 09-0077; JCPDS card No. 072-0713; JCPDS card No. 074-1301; Koutsopoulos, S. Synthesis and Characterization of Hydroxyapatite Crystals: A Review Study on the Analytical Methods. *J. Biomed. Mater. Res.* 2002, 62 (4), 600–612; e) Karampas, L.A., Kontoyannis, C.G., Characterization of calcium phosphates mixtures. *Vib. Spec.* 2013, 64, 126–133.

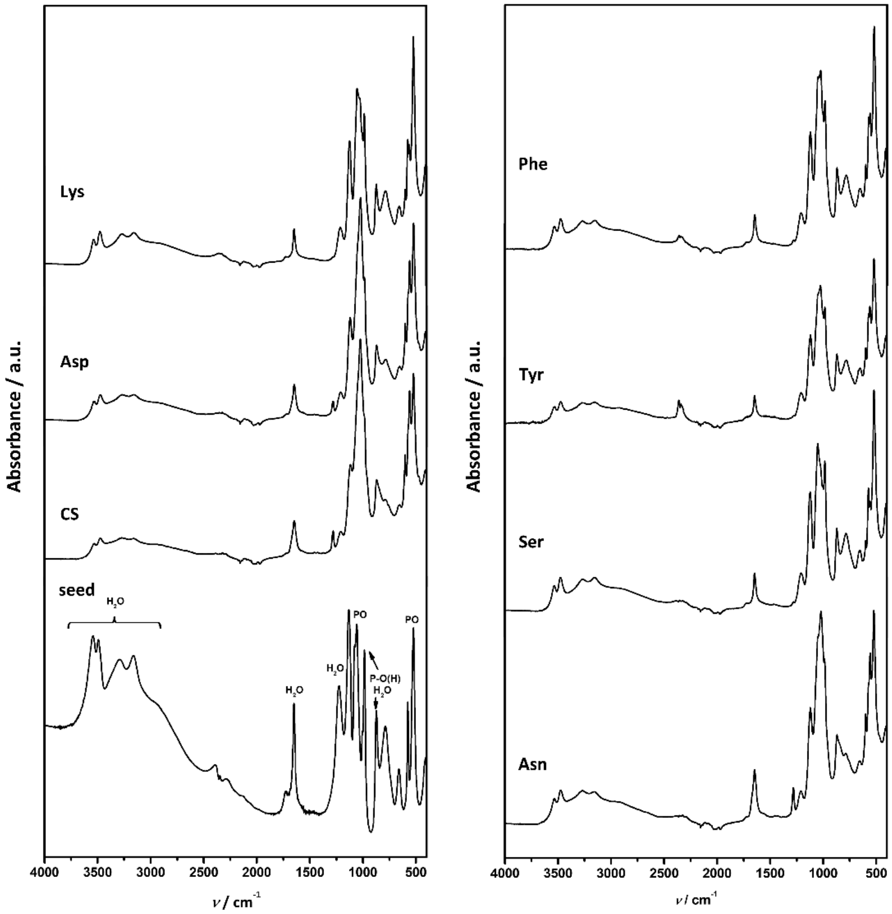

Figure S6. FTIR spectra of calcium hydrogenphosphate dihydrate (DCPD) seed crystals and precipitates formed in the control system (CS) and in the presence of different amino acids after 60 min aging time in the system $c(CaCl_2) = c(Na_2HPO_4) = 4.198$ mmol dm^{-3}, c(NaCl) = 0.148 mol dm^{-3}, m(seed DCPD) = 1 mg, γ(AA) = 10 ppm except γ(Phe) = 5 ppm t = 25 °C, $pH_{initial} = 7.4$, magnetic stirring. Asp—L–aspartic acid, Tyr—L–tyrosine, Asn—L–asparagine, Lys—L–lysine, Ser—L–serine, Phe—L–phenylalanine

Table S8. Assignment of IR bands in FTIR spectra of calcium hydrogenphosphate dihydrate (DCPD) seed crystals and precipitates formed in the control system (CS) and in the presence of different amino acids after 60 min aging time in the system $c(CaCl_2) = c(Na_2HPO_4)$ = 4.198 mmol dm^{-3}, c(NaCl) = 0.148 mol dm^{-3}, m(seed DCPD) = 1 mg, γ(AA) = 10 ppm except γ(Phe) = 5 ppm t = 25 °C, $pH_{initial}$ = 7.4, magnetic stirring. Asp—L–aspartic acid, Tyr—L–tyrosine, Asn—L–asparagine, Lys—L–lysine, Ser—L–serine, Phe—L–phenylalanine.

Wavenumber/cm^{-1}								Band Assignment
**Seed**	**CS**	**Asp**	**Lys**	**Asn**	**Ser**	**Tyr**	**Phe**	
3548	3542	3530	3538	3539	3533	3540	3537	O-H stretching of water
3493	3480	3480	3478	3475	3480	3473	3481	O-H stretching of water
3291	3265	3260	3274	3271	3269	3275	3267	O-H stretching of water
3149	3156	3150	3157	3161	3148	3159	3154	O-H stretching of water
						2362	2362	PO-H stretching
2388			2341			2343	2337	PO-H stretching
1723			1721		1721		1721	O-H bending of water
1636	1644	1650	1647	1651	1644	1645	1647	O-H bending of water
	1282	1282	1279	1277	1282	1283	1280	O-H bending of HPO_4^{2-}
1231								O-H in plane bending
	1206	1211	1215		1207	1207	1210	O-H in plane bending
1131								PO stretching
	1118	1122	1123	1121	1122	1121	1121	ν_{3a} *a*symmetric PO stretching
1055			1051		1054		1045	Asymmetric PO stretching
	1025	1019	1023	1020	1034	1022	1029	ν_{3a} asymmetric PO stretching
987		984	986		982	986	985	ν_1 symmeric P-O(H) stretching
				963	960	959		P-O(H) stretching
872	869	869	872	865	874	872	872	P-O(H) stretching
783	787	780	784	789	787	780	783	H_2O libration
657	655	654	655	649	655	650	653	H_2O libration
	603	599	600	600	600	599	599	ν_{4a} PO bending
572					572	575	575	ν_{4c} PO bending (O-P-O)
562	560	555	559	556		555	564	ν_{4c} PO bending (O-P-O)
525	523	516	520	520	516	523	523	ν_2 PO bending (O-P-O)

[a] Karampas, I. A.; Kontoyannis, C. G. Characterization of Calcium Phosphates Mixtures. *Vib. Spec.* **2013**, *64*, 126–133; [b] Xu, J.; Butler, I. S.; Gilson, D. F. R. FT-Raman and High-Pressure Infrared Spectroscopic Studies of Dicalcium Phosphate Dihydrate ($CaHPO_4 2H_2O$) and Anhydrous Dicalcium Phosphate ($CaHPO_4$). *Spec. Acta A:* **1999**, *55* (14), 2801–2809, Petrov, I.; Šoptrajanov, B.; Fuson, N.; Lawson, J.R. Infra-red investigation of dicalcium phosphates. *Spec. Acta* **1967**, *23A*, 2637-2646.

Table S9. Intensity of selected phosphate bands and their ratios for calcium hydrogenphosphate dihydrate (DCPD) seed crystals and precipitates formed in the control system (CS) and in the presence of different amino acids in the system $c(CaCl_2) = c(Na_2HPO_4) = 4.198$ mmol dm^{-3}, c(NaCl) = 0.148 mol dm^{-3}, m(seed DCPD) = 1 mg, γ(AA) = 10 ppm except γ(Phe) = 5 ppm t = 25 °C, $pH_{initial}$ = 7.4, magnetic stirring. Asp—L–aspartic acid, Tyr—L–tyrosine, Asn—L–asparagine, Lys—L–lysine, Ser—L–serine, Phe—L–phenylalanine. A1—intensity of P-O(H) stretching at around 872 cm^{-1}, A2—ν_{4c} *PO* bending (O-P-O) at around 560 cm^{-1}, A3—ν_2 PO bending (O-P-O) at around 525 236 cm^{-1}.

	**60 min Aging Time**					
	**A1**	**A2**	**A3**	**A1:A2**	**A1:A3**	**A2:A3**
seed	0.073	0.164	0.332	0.45	0.22	0.49
CS	0.041	0.104	0.116	0.39	0.35	0.90
Asp	0.049	0.122	0.162	0.40	0.30	0.75
Lys	0.062	0.081	0.211	0.77	0.29	0.38
Asn	0.051	0.045	0.141	1.13	0.36	0.32
Ser	0.048	-	0.153	-	0.31	-
Tyr	0.021	0.034	0.058	0.62	0.36	0.59
Phe	0.046	0.076	0.157	0.61	0.29	0.48
	**240 min Aging Time**					
CS	0.027	0.239	-	0.11	-	-
Asp	0.034	0.179	-	0.19	-	-
Lys	0.041	0.192	0.142	0.21	0.29	1.35
Asn	0.036	0.169	0.094	0.21	0.38	1.80
Ser	0.016	0.141	0.046	0.11	0.35	3.07
Tyr	0.032	0.199	0.107	0.16	0.30	1.86
Phe	0.034	0.195	0.116	0.17	0.29	1.68

Table S10. Assignment of reflections in PXRD diffractograms of calcium hydrogenphosphate dihydrate (DCPD) seed crystals and precipitates formed in the control system (CS) and in the presence of different amino acids after 240 min aging time in the system $c(CaCl_2) = c(Na_2HPO_4) = 4.198$ mmol dm^{-3}, c(NaCl) = 0.148 mol dm^{-3}, m(seed DCPD) = 1 mg, γ(AA) = 10 ppm except γ(Phe) = 5 ppm t = 25 °C, $pH_{initial}$ = 7.4, magnetic stirring. Asp—L–aspartic acid, Tyr—L–tyrosine, Asn—L–asparagine, Lys—L–lysine, Ser—L–serine, Phe—L–phenylalanine.

**2θ/°**								**hkl**
**Seed**	**CS**	**Asp**	**Lys**	**Asn**	**Ser**	**Tyr**	**Phe**	
	4.74	4.80		4.62	4.71			OCP (100)
	9.56			9.54	9.48	9.41		OCP $(\bar{1}10)$
11.58		11.61	11.61	11.64		11.64	11.60	(020)
20.91		20.96	20.90	20.91		20.83	20.96	(021)
23.31								(040)
25.98	25.87	25.87	25.94	25.95	25.75	26.03	25.91	$(\bar{1}31)$
				28.12	28.30			
29.22		29.29	29.27	29.30		29.30	29.36	(041)
30.52		30.53	30.42	30.50		30.44	30.51	$(\bar{2}21)$
31.84								(200)
	31.92	31.85	31.54	31.87	31.84	31.69	31.56	CaDHA
34.08		34.15	34.11	34.23		34.23	34.21	$(\bar{2}20)$
36.96		36.91	36.91				36.65	(022)
		39.32		39.18	39.27			(212)
39.69								(220)
				40.64				
41.63		41.63	41.65	41.58				(151)
42.05							42.09	$(\bar{2}42)$
42.97				43.06				$(\bar{1}52)$
43.29								$(\bar{3}11)$
44.71								(170)
45.19			45.22				45.15	$(\bar{1}71)$
45.96								(112)

	46.52				46.54			CaDHA
47.99								(080)
48.46			48.41					$(\bar{2}60)$
49.14								(132)
	49.44			49.58	49.50			CaDHA
		50.03				50.05	50.08	CaDHA
50.13			50.13					(241)
50.83								(062)
51.42								(081)
53.53								$(18\bar{1})$
	53.62	53.48	53.46	53.45	53.39	53.55	53.39	CaDHA
55.27								$(25\bar{3})$
56.52								(181)
58.79								(082)
59.54								$(20\bar{4})$

Assignment made according to: JCPDS card No. 09-0077; JCPDS card No. 074-1301; JCPDS card No. 072-0713; Koutsopoulos, S. Synthesis and Characterization of Hydroxyapatite Crystals: A Review Study on the Analytical Methods. *Journal of Biomedical Materials Research* 2002, 62 (4), 600–612; Karampas, L.A., Kontoyannis, C.G., Characterization of calcium phosphates mixtures, *Vib. Spec.* **2013**, *64*, 126–133.

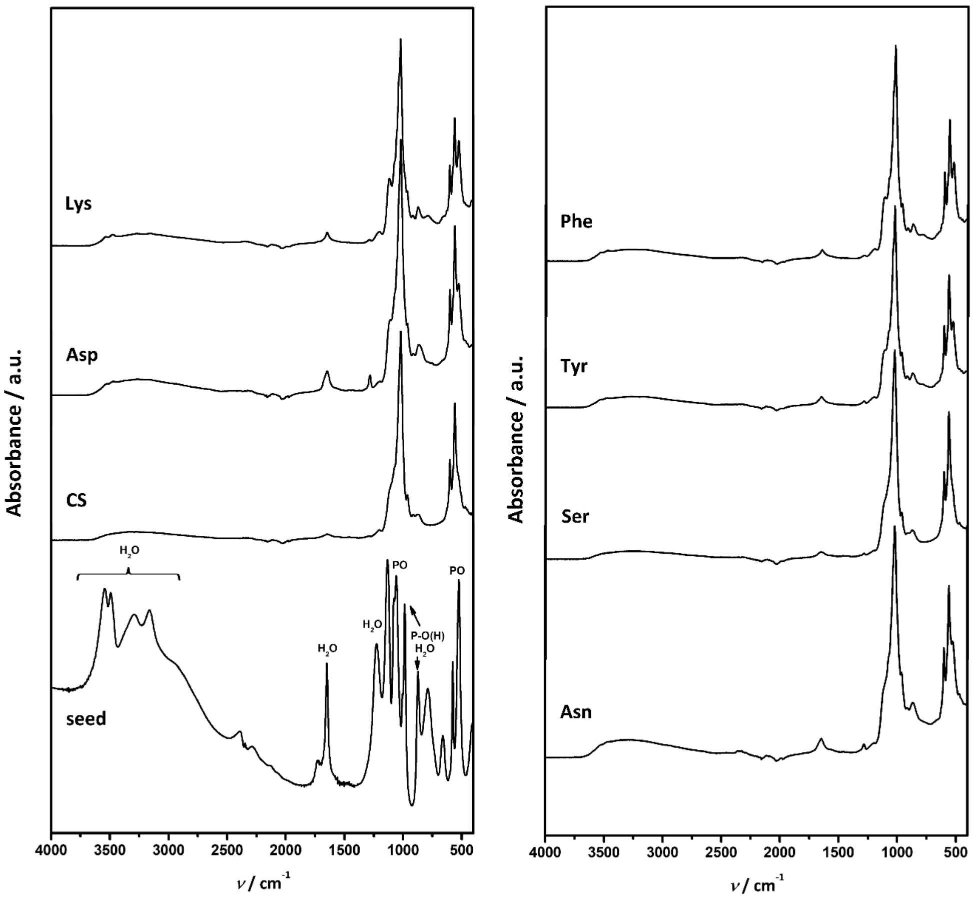

Figure S7. FTIR spectra of calcium hydrogenphosphate dihydrate (DCPD) seed crystals and precipitates formed in the control system (CS) and in the presence of different amino acids after 240 min in the system $c(CaCl_2) = c(Na_2HPO_4) = 4.198$ mmol dm^{-3}, c(NaCl) = 0.148 mol dm^{-3}, m(seed DCPD) = 1 mg, γ(AA) = 10 ppm except γ(Phe) = 5 ppm t = 25 °C, $pH_{initial}$ = 7.4, magnetic stirring. Asp—L–aspartic acid, Tyr—L–tyrosine, Asn—L–asparagine, Lys—L–lysine, Ser—L–serine, Phe—L–phenylalanine.

Table S11. Assignment of IR bands in FTIR spectra of calcium hydrogenphosphate dihydrate (DCPD) seed crystals and precipitates formed in the control system (CS) and in the presence of different amino acids after 240 min aging time in the system $c(CaCl_2) = c(Na_2HPO_4)$ = 4.198 mmol dm^{-3}, c(NaCl) = 0.148 mol dm^{-3}, m(seed DCPD) = 1 mg, γ(AA) = 10 ppm except γ(Phe) = 5 ppm t = 25 °C, $pH_{initial}$ l = 7.4, magnetic stirring. Asp—L−aspartic acid, Tyr—L−tyrosine, Asn—L−asparagine, Lys—L−lysine, Ser—L−serine, Phe—L−phenylalanine.

Wavenumber/cm^{-1}								Band Assignment
**Seed**	**CS**	**Asp**	**Lys**	**Asn**	**Ser**	**Tyr**	**Phe**	
	3651–2574	3679–2570		3679–2565	3646–2615	3654–2572	3648–2591	water vibration[a]
3548			3530					O-H stretching of water
3493			3471					O-H stretching of water
3291								O-H stretching of water
3149								O-H stretching of water
2388								PO-H stretching
			2343					PO-H stretching
1723								O-H bending of water
1636	1650	1651	1646	1651	1647	1645	1642	O-H bending of water
		1282	1284	1280	1282	1283	1288	O-H bending of HPO42-
1229								O-H in plane bending
	1197		1206		1199	1194	1202	O-H in plane bending
1131								PO stretching
			1117			1113	1109	PO stretching
1055								ν_{3a} asymmetric PO stretching
	1022	1022	1021	1020	1023	1023	1023	Asymmetric PO stretching
987								ν_1 symmeric P-O(H) stretching
	963	965		963	959	964	959	P-O(H) stretching
			916			912	915	P-O(H) stretching
872	865	861	865	861	865	863	869	P-O(H) stretching
783			784					H_2O libration
657								H_2O libration
	601	601	600	600	601	601	602	ν_{4a} PO bending
572								ν_{4c} PO bending (O-P-O)
562	560	560	559	560	559	555	560	ν_{4c} PO bending (O-P-O)
525			523	526	526	520	526	ν_2 PO bending (O-P-O)

[a] Karampas, I. A.; Kontoyannis, C. G. Characterization of Calcium Phosphates Mixtures. *Vib. Spec.* **2013**, *64*, 126–133; [b] Xu, J.; Butler, I. S.; Gilson, D. F. R. FT-Raman and High-Pressure Infrared Spectroscopic Studies of Dicalcium Phosphate Dihydrate (CaHPO42H2O) and Anhydrous Dicalcium Phosphate (CaHPO4). *Spec. Acta A:* **1999**, *55* (14).
